# Wolbachia Modulates Lipid Metabolism in Aedes albopictus Mosquito Cells

**DOI:** 10.1128/AEM.00275-16

**Published:** 2016-05-02

**Authors:** Jennifer C. Molloy, Ulf Sommer, Mark R. Viant, Steven P. Sinkins

**Affiliations:** a Nuffield Department of Medicine and Department of Zoology, University of Oxford, Oxford, United Kingdom; b NERC Biomolecular Analysis Facility—Metabolomics Node, School of Biosciences, University of Birmingham, Birmingham, United Kingdom; c Biomedical and Life Sciences, Lancaster University, Lancaster, United Kingdom; University of Michigan

## Abstract

Certain strains of the intracellular endosymbiont Wolbachia can strongly inhibit or block the transmission of viruses such as dengue virus (DENV) by Aedes mosquitoes, and the mechanisms responsible are still not well understood. Direct infusion and liquid chromatography-Fourier transform ion cyclotron resonance (FT-ICR) mass spectrometry-based lipidomics analyses were conducted using Aedes albopictus Aa23 cells that were infected with the *w*Mel and *w*MelPop strains of Wolbachia in comparison to uninfected Aa23-T cells. Substantial shifts in the cellular lipid profile were apparent in the presence of Wolbachia. Most significantly, almost all sphingolipid classes were depleted, and some reductions in diacylglycerols and phosphatidylcholines were also observed. These lipid classes have previously been shown to be selectively enriched in DENV-infected mosquito cells, suggesting that Wolbachia may produce a cellular lipid environment that is antagonistic to viral replication. The data improve our understanding of the intracellular interactions between Wolbachia and mosquitoes.

**IMPORTANCE** Mosquitoes transmit a variety of important viruses to humans, such as dengue virus and Zika virus. Certain strains of the intracellular bacterial genus called Wolbachia found in or introduced into mosquitoes can block the transmission of viruses, including dengue virus, but the mechanisms responsible are not well understood. We found substantial shifts in the cellular lipid profiles in the presence of these bacteria. Some lipid classes previously shown to be enriched in dengue virus-infected mosquito cells were depleted in the presence of Wolbachia, suggesting that Wolbachia may produce a cellular lipid environment that inhibits mosquito-borne viruses.

## INTRODUCTION

Wolbachia endosymbionts are common intracellular, maternally inherited bacteria that manipulate host reproduction. The most common form of manipulation is cytoplasmic incompatibility (CI), patterns of crossing sterile individuals that can provide a reproductive advantage to females carrying the bacteria, allowing rapid population spread and maintenance at high frequency (1). In certain host-strain combinations, in particular where high bacterial densities are reached, Wolbachia can significantly reduce the transmission of some of the most important mosquito-borne pathogens of humans, including dengue (DENV) and Chikungunya viruses and malaria and filarial nematode parasites (2–10). The *w*Mel strain of Wolbachia pipientis has been used for field trials in Aedes aegypti mosquitoes, having reached and remained at a very high population frequency (11, 12), and wild carriers still show greatly reduced DENV susceptibility several years later (13). The mechanisms behind the pathogen inhibition phenotype remain uncertain; upregulation of innate immune genes has been observed and can contribute to the phenotype (2–5, 9, 14) but is apparently not required for efficient viral transmission blocking (7, 8, 15).

The cellular lipidome often undergoes major changes in the presence of pathogens, as seen for mosquito cells infected with DENV; inhibition of fatty acid synthase at 4 to 12 h after DENV infection results in greatly reduced viral titers (16). In mammalian cells, autophagy-mediated release of triglyceride fatty acids from lipid droplets is required for DENV replication (17–21). Cholesterol can also play an important role in arbovirus entry to mammalian cells (22–25), viral envelope formation (17), and exit from the cell (26). Lipid metabolism and autophagy are not only implicated in pathogen-mosquito interactions but are likely to be critical to the Wolbachia-host relationship. The streamlined genome of Wolbachia lacks synthesis pathways for fatty acids and cholesterol (27), so Wolbachia-host symbioses provide a likely arena for resource competition that could in turn impact viral refractoriness. Insects can synthesize fatty acids and other lipid classes but are also sterol auxotrophs (28), increasing the potential for competition over dietary cholesterol. Cholesterol modulation by Wolbachia has already been suggested to play a functional role in DENV infection in *w*Mel-infected Drosophila melanogaster flies, where increased dietary cholesterol reduced viral refractoriness (29), but any role in mosquitoes remains an open question.

The influence of Wolbachia on broader host lipid metabolism remains unknown, as few data have been published, but there are potential mechanistic links. Autophagy is known to alter lipid profiles and has been shown to reduce Wolbachia density if upregulated, leading to the suggestion that Wolbachia might modulate the process in hosts (30). As DENV requires autophagy for replication, this is interesting in the context of *w*Mel-infected D. melanogaster flies, which are protected against several RNA viruses but not the DNA virus insect iridovirus VI (31), which replicates in the nucleus so likely does not require autophagy (32). An investigation of heritability and interpopulation differences in D. melanogaster lipid profiles found that Wolbachia abundance correlated with a gain of odd-chained fatty acids in females and an increase in phosphatidylserines, a class of phospholipids which are responsible for signaling that results in the clearing of apoptotic cells, which the authors suggested could arise from the induction of stress and apoptosis by Wolbachia (33). No such data are available for Wolbachia in mosquitoes. In order to broadly identify candidate lipid classes and lipid pathways modulated in Wolbachia-host metabolic interactions, an untargeted lipidomics analysis was performed on Aedes albopictus Aa23 cell lines containing the *w*Mel or *w*MelPop strains in comparison with Wolbachia-free Aa23-T cells. A. albopictus is an invasive mosquito species (34) that is important in the transmission of DENV and chikungunya virus (CHIKV), both of which impose high and increasing global disease burdens (35, 36), but neither virus was transmitted when the naturally occurring *w*AlbA and *w*AlbB Wolbachia strains were replaced with *w*Mel from Drosophila melanogaster (7, 8).

For this purpose, we used high-resolution Fourier transform ion cyclotron resonance (FT-ICR)-based nontargeted lipidomics applying both liquid chromatography-mass spectrometry (LC-MS) and nanoelectrospray direct infusion mass spectrometry (DIMS). While the lipid LC-MS approach is inherently suited to reduce ion suppression and enable more rigorous annotation of *m/z* signals due to retention time information (37), the DIMS method can detect species outside the optimal range of the LC gradient, reduces ion suppression by using nanoelectrospray ionization, and maximizes sensitivity and mass accuracy by acquiring data in a series of selected ion monitoring windows (38, 39).

## MATERIALS AND METHODS

### Aedes albopictus cell lines and Wolbachia density analysis.

The Aa23 mosquito cell line (40), derived from A. albopictus eggs and cured of its native Wolbachia infection using antibiotics (Aa23-T), and two derived lines that we had transinfected with Wolbachia strains from Drosophila to create Aa23.*w*Mel and Aa23.*w*MelPop (41) were grown in 25-cm^2^ or 75-cm^2^ flasks kept at 27°C in Schneider's medium (Promo-Cell) with 10% fetal bovine serum (FBS) and 1% penicillin-streptomycin (Gibco). The standard passage time was 5 to 7 days. Wolbachia density was analyzed by quantitative real-time PCR (qPCR) via absolute quantification against a dilution curve of a vector containing single copies of the homothorax (HTH) gene (42) and Wolbachia surface protein (*wsp*). Primer sequences were as follows: Mos qHTH F, 5′-TGGTCCTATATTGGCGAGCTA-3′; Mos qHTH R, 5′-TCGTTTTTGCAAGAAGGTCA-3′; qWSP all-F, 5′-ATCTTTTATAGCTGGTGGTGGT-3′ (3); qWSP all-R, 5′-AAAGTCCCTCAACATCAACCC-3′.

DNA was extracted with CTAB (cetyltrimethylammonium bromide) buffer as previously described (41), 100 ng was added to a 10-μl qPCR mixture using SYBR green Express qPCR supermix universal (Life Technologies) per the manufacturer's instruction, and qPCR was performed in an MJ Research PTC-200 DNA Engine using a Chromo4 detection system and Opticon Monitor software v3.1 (Bio-Rad Laboratories). Cycling conditions were 95°C for 4 min, followed by 40 cycles at 95°C for 15 s and then 59°C for 30 s. The number of Wolbachia genomes per host genome was determined from a linear model fitted to the known plasmid standard curve values as previously described (41).

### Lipid extraction from Aa23 cell lines.

Aa23-T, Aa23.*w*Mel, and Aa23.*w*MelPop cells were grown in 25-cm^2^ flasks in 5 ml of medium with the passage of 1 ml to 5 ml of new medium every 5 days. Eight flasks per line were independently passaged for at least 5 weeks before lipid and DNA extraction. Cells were quenched 72 h after the final passage; fetal bovine serum (FBS) from the same batch was used throughout, and cell growth was monitored to ensure that cells were harvested in the late logarithmic phase at 80 to 90% confluence. A 1.6-ml volume of quenching solution (60:40, high-performance LC [HPLC]-grade methanol–water [MeOH-H_2_O; Sigma-Aldrich]) per flask was precooled in each 2-ml tube on dry ice to approximately −40°C. Cells were scraped into the flask medium with minimum disruption, and the cell suspension was transferred to a 15-ml tube for centrifugation at room temperature for 5 min at 1,000 relative centrifugal force (RCF). A 500-μl cell suspension was then retained for DNA extraction. The supernatant was removed by pouring, and cell pellets were resuspended in residual medium by gentle pipetting. A 200-μl volume of this cell suspension was added to 1.6 ml of quenching solution at −40°C and mixed immediately by inversion. Samples were centrifuged in the prechilled rotor at −9°C and 2,500 RCF for 5 min. A maximum of 10 samples were processed at a time to ensure that all were kept below 0°C. The quenching solution was removed with a glass Pasteur pipette, and the tube was weighed to estimate cell mass before being frozen at −80°C and was retained at this temperature until extraction.

For lipid extraction, HPLC-grade MeOH (EMD Millipore), H_2_O, and chloroform (Sigma-Aldrich) were cooled on ice, and 8 μl of MeOH/mg pellet mass and 7.2 μl of H_2_O/mg were added to each sample tube. Tubes were vortexed for 30 s and returned to ice. An 8-μl/mg concentration of chloroform was added to 1.8-ml glass vials, and after pulse vortexing, the pellet and MeOH-H_2_O mixture were transferred using a glass Pasteur pipette. Samples were vortexed and incubated on ice for 10 min before centrifugation at 1,500 RCF for 15 min at 4°C. Biphasic samples were left at room temperature for 5 min before the upper layer containing polar metabolites was removed using a Hamilton syringe. A 270-μl volume of nonpolar lipid-containing lower layer was then transferred to a 1.8-ml glass tube and stored overnight at −80°C. Seven Aa23-T samples and six samples from each infected line were used. Forty microliters of each sample was removed, and these samples were pooled to form a quality control (QC) sample before all samples were dried under nitrogen, taken back up in 270 μl chloroform, and stored at −80°C until analysis.

### DI FT-ICR MS and data processing.

Negative-ion-mode direct-infusion (DI) FT-ICR MS was conducted using a hybrid 7-T linear ion trap FT-ICR mass spectrometer (LTQ FT Ultra; Thermo Fisher Scientific) equipped with an Advion Triversa chip-based direct-injection nanoelectrospray ion source (Advion Biosciences). Samples were diluted 1:4 in 5 mM ammonium acetate (from a 1 M stock in methanol) in methanol-chloroform (3:1). Technical triplicates were run in controlled randomized order. Data were acquired from *m/z* 120 to 1,200 in 12 wide SIM (selected ion monitoring) windows (43). Additional compound identification (direct infusion tandem MS [DI-MS/MS] and multiple-stage MS [MS^n^]) was performed on the same instrument.

Data processing was performed using NBAF-B in-house scripts in MatLab, the SIMStitch pipeline (44). During “replicate filtering,” peaks were retained if they were found in two out of three technical replicates. Subsequently a “blank filter” required that biological signals be two times the intensity of background signals to be retained. Sample filtering with a 2-ppm mass error and a 75% filter resulted in a matrix containing 2,044 peaks (*m/z* values). Normalization was performed using the probabilistic quotient normalization (PQN) method, and missing values were imputed in the resulting data matrix using the *k*-nearest neighbors (KNN) algorithm. A generalized logarithm-transformed version of this normalized, missing value-imputed matrix was generated (based on the technical variance of the QC samples) for use only in the multivariate statistical analysis.

### LC FT-ICR MS and data processing.

Reversed-phase high-performance liquid chromatography (RPLC) FT-ICR MS-based analysis of the lipids was carried out on a Thermo Scientific Dionex Ultimate 3000 system connected to the same mass spectrometer as described above. Samples were dried, and each was taken up in the original volume of solvent A (5 mM ammonium acetate, 5% isopropanol in water)-solvent B (5 mM ammonium acetate, 5% isopropanol, 5% water in acetonitrile) (1:1) and centrifuged for 10 min at 4°C and 4,000 RCF. Eight microliters of each sample was injected onto a Dionex Acclaim C_30_ column (Thermo Scientific; 2.1 by 150 mm, 3-μm particles) and separated at 40°C with a flow rate of 200 μl/min and a gradient from 100% solvent A to 100% solvent B to 100% solvent C (5 mM ammonium acetate, 5% water in isopropanol) (see Table S11 in the supplemental material). The gradient was designed to thoroughly wash out polar compounds before lipid analysis. One LC FT-ICR MS analysis was performed per sample. Data were acquired in negative-ion mode from *m/z* 120 to 1,200 at a nominal resolution of 50,000 in centroid mode. A QC sample was pooled from all biological samples and analyzed repeatedly at the start and end of and equidistantly throughout the sequence. Two further QC sample runs were performed using data-dependent acquisition (MS/MS) of the most intense ions in the negative- and positive-ion modes, respectively.

RPLC FT-ICR MS lipidomics data were initially converted into netcdf (.cdf) format using Xcalibur 2.1 and processed using an R-based XCMS/Camera script (see Table S12 in the supplemental material) (45, 46) to generate an intensity matrix with a list of peak retention times. The intensity matrix was imported into the SIMStitch pipeline immediately after the replicate filter and hence included blank filtering, sample filtering, PQN normalization, KNN missing-value imputation, and the generalized logarithm transformation, as for DIMS processing above. The sample filter was set to 75%, since no technical replicate filtering was applied.

### Statistical analysis and identification of metabolites.

Principal-component analysis (PCA) was used initially to assess the overall metabolic differences between the sample groups in an unbiased manner, using the PLS_Toolbox (version 7.8.2; Eigenvector Research, Manson, WA, USA) within Matlab (version 8.1; The MathWorks, Natick, MA, USA). Supervised multivariate analyses were performed using partial least-squares discriminant analysis (PLS-DA) using the PLS Toolbox, with internal cross validation (venetian blinds) and permutation testing (1,000 tests each) using an in-house Matlab script, which created a single variable importance list from the latent variable (LV) vectors and forward selected these variables for an optimized PLS model. Univariate statistical analyses were used to confirm the significance of changes in individual mass spectral signals. Specifically, analysis of variance (ANOVA) was conducted using an in-house MatLab script with a false discovery rate of 5% to correct for multiple hypothesis testing (47).

For both lipid data sets, peaks were annotated and putative empirical formulae were calculated using MI-Pack software (48), with searches of the KEGG (http://www.genome.jp/kegg/) and Lipid Maps (http://www.lipidmaps.org/) databases (49–51). Additional MS/MS data were acquired as described above and analyzed in Xcalibur 3.0.63 (Thermo Fisher Scientific). Lipid classes were identified, with initial focus on the most intense species, by analysis of collected MS/MS data, isotopic patterns, and, for LC-MS, the expected relative retention times.

The data set is available in the MetaboLights database at http://www.ebi.ac.uk/metabolights/MTBLS210, and the R code used to generate the figures is available at https://github.com/jcmolloy/lipidomicspaper.

## RESULTS

**FIG 1:**
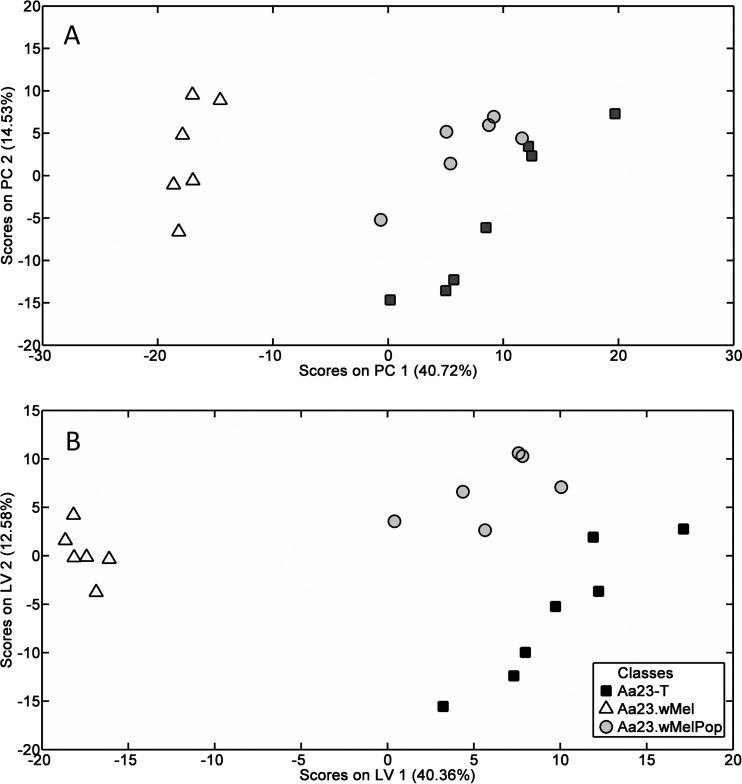
Direct infusion mass spectrometry (DIMS) shows distinct lipid profiles in Wolbachia-infected versus uninfected A. albopictus Aa23 cell lines. (A) Principal-component analysis (PCA) plot showing segregation of total lipid profiles measured using DIMS. (B) Partial least-squares discriminant analysis (PLS-DA) of DIMS data. Data points represent six or seven biological replicates from Aa23-T, Aa23.*w*Mel, and Aa23.*w*MPop cell lines, and 2,044 signals from the negative-ion DIMS analysis were analyzed.

Both DIMS and LC-MS were conducted on lipid extracts from Aa23-T (Wolbachia-uninfected), Aa23.*w*Mel, and Aa23.*w*MelPop (*w*Mel-infected and *w*MelPop-infected, respectively) cells. Negative-ion DIMS analysis of the lipid extracts resulted in 2,044 peaks, which clustered into distinct lipid profiles for the three groups, distinguished by PCA and PLS-DA. There were clear separations of Aa23-T and Aa23.*w*Mel along principal component 1 (PC1) and between Aa23-T and Aa23.*w*MelPop along the same axis but to a lesser degree (Fig. 1A). PLS-DA results showed that Aa23.*w*MelPop was separated from the other lines along latent variable 1 (LV1), while separation between Aa23-T and Aa23.*w*Mel was mostly along LV2 and, to a lesser extent, LV1 (Fig. 1B). Internal cross validation testing of the PLS-DA model showed reasonable classification errors and significant *P* values for all three treatment groups in the PLS-DA model (see Table S1 in the supplemental material).

**FIG 2:**
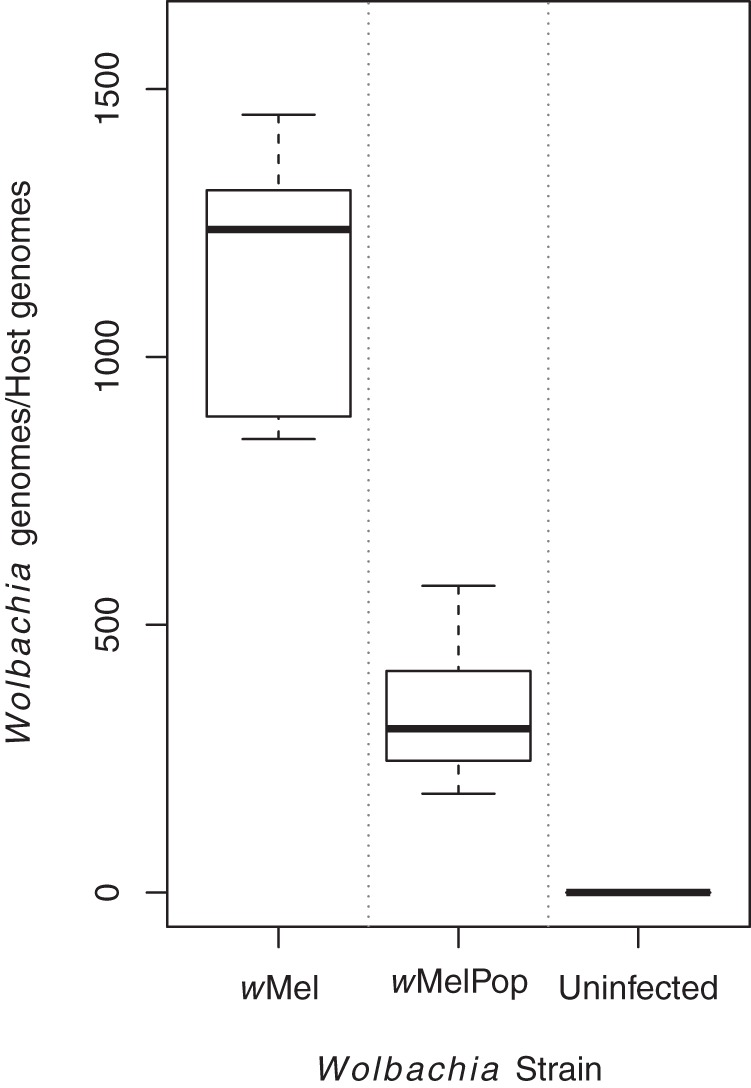
Wolbachia density in Aa23.*w*Mel was over three times higher than that in Aa23.*w*MelPop cell line. The ratios of Wolbachia genome copies to A. albopictus genome copies detected in Aa23.wMel and Aa23.wMelPop cell lines are shown. Genome copies were determined by absolute quantification using a standard curve of plasmid DNA containing the genes of interest. Results are from eight replicates per cell line, independently passaged for 5 weeks, with two replicates of uninfected Aa23 used as a negative control.

**FIG 3:**
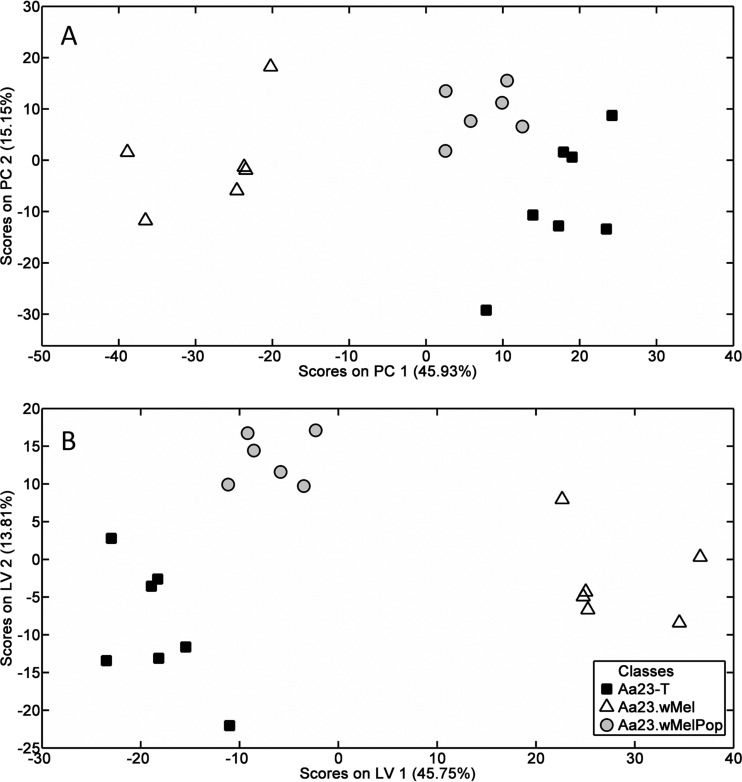
Liquid chromatography-mass spectrometry (LC-MS) shows distinct lipid profiles in Wolbachia-infected versus uninfected A. albopictus Aa23 cell lines. (A) Principal-component analysis (PCA) plot showing segregation of total lipid profile derived from LC-MS. (B) Partial least-squares discriminant analysis (PLS-DA) of resulting data. Points represent six or seven biological replicates from Aa23, Aa23.*w*Mel, and Aa23.*w*MelPop cell lines, and 4,736 signals from the LC-MS analysis were analyzed.

Differences were most profound between Aa23-T and Aa23.*w*Mel, which was unexpected, as *w*Mel infections are typically maintained at a lower density than *w*MelPop infections. However, densities can fluctuate in cell lines (52), and qPCR of DNA samples taken from six separate culture flasks per line concurrently with the lipid extraction revealed that at the time of sampling, the mean Wolbachia density was >3-fold higher in Aa23.*w*Mel than in Aa23.*w*MelPop. The recorded densities of 1,147 ± 236 *w*Mel genomes per cell versus 336 ± 127 *w*MelPop genomes per cell (Fig. 2) are in line with DENV-blocking densities in cell lines reported previously (53). An LC-MS analysis was performed to complement the nanoelectrospray DIMS measurements. Analysis of 4,736 signals supported the pattern of separation along the principal components and latent variables observed in the analysis of the DIMS data but showed clearer segregation of the Wolbachia-infected and -uninfected cell lines (Fig. 3; see Table S2 in the supplemental material). Further analysis was thus undertaken focusing mostly on the LC-MS data set, which contained 2,794 signals that differed significantly in intensity between cell lines (ANOVA, *q* < 0.05) while the DIMS data set contained 513.

For both data sets, PLS-DA was also performed comparing each set of two groups, and after reclassification of the Aa23.*w*Mel and Aa23.*w*MelPop groups into one “infected” group. All of the PLS-DA models had *P* values of <5% (from permutation testing) and classification errors of <20% (from internal cross validation; see Tables S3 to S10 in the supplemental material), with the DIMS model comparing Aa23-T and Aa23.*w*MelPop (see Table S5) being weaker than the others.

The 513 differentially regulated DIMS signals resulted in 290 putative annotations (see Table S15 in the supplemental material), and the 2,794 in the LC-MS data set resulted in 1,003 putative annotations (see Table S16) by use of the MI-Pack (48) and both the Lipid Maps and KEGG databases. This analysis clearly revealed which lipid classes differed between the three groups of samples. Additional species were annotated manually, especially nonstandard adducts [NO_3_^−^ and Na(OAc)_2_^−^, where OAc is acetate], and others were manually curated, taking into account the measured MS/MS data, relative signal intensities, and in the case of LC-MS, retention times. For the majority of species, no MS/MS data were collected, with the larger lipids annotated by summing up their constituent acyl and alkyl chains [e.g., Cer(d18:1/18:0) is represented as Cer 36:1].

### Sphingolipids.

**FIG 4:**
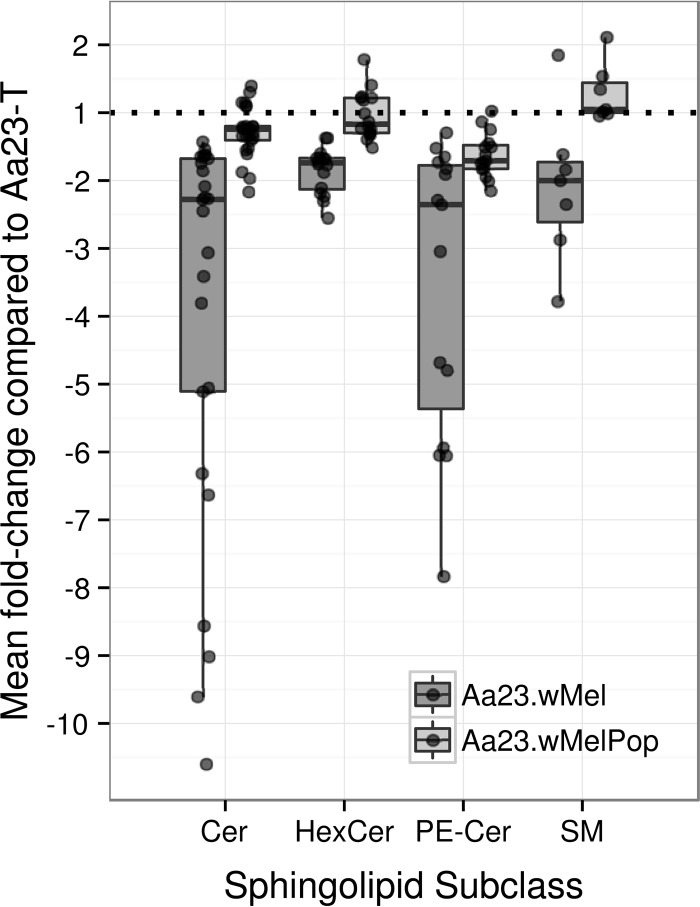
Sphingolipids show a mean decrease in Wolbachia-infected A. albopictus Aa2 cell lines. A box plot shows mean fold changes in sphingolipid species abundance in the Wolbachia-infected A. albopictus cell lines Aa23.*w*Mel and Aa23.*w*MelPop relative to that of uninfected Aa23-T. Negative fold changes reflect the increase of the signal in the uninfected cells compared to that of the infected cells. LacCer is not included due to the small number of data points for this lipid class. Cer, ceramide, [M+OAc]^−^ ion series; HexCer, hexosylceramide, [M-H]^−^ ion series; PE-Cer, phosphatidylethanolamine ceramide, [M-H]^−^ ion series; SM, sphingomyelin, [M+OAc]^−^ ion series.

Many of the more intense signals in the spectra are derived from sphingolipids, which play a major structural role in plasma membranes in addition to being involved in signaling and a range of other functions within the cell (54–57). However, there has been little reported research on the biological role of specific sphingolipids in mosquitoes. Ceramides appear to be the sphingolipid class most affected by Wolbachia infection (Fig. 4). For example, in the LC-MS PLS-DA model comparing Aa23-T and Aa23.*w*Mel cell lines, six different ceramides were featured in the top 10 annotated lipid signals ranked by variable importance (a vector calculated from the model's latent variables) (see Materials and Methods and the supplemental material), with an additional two ^13^C isotopic ceramide signals. Four of these ceramides also were featured in the top 10 most important ranked lipid signals for Aa23-T versus Aa23.*w*MelPop and for control versus infected (combined Aa23.*w*Mel and Aa23.*w*MelPop).

In the DIMS data set, 80 signals were initially annotated as ceramides by MI-Pack (as the adducts [M-H]^−^, [M+OAc]^−^, M+Cl]^−^, [M+^37^Cl]^−^, and [M+K-2H]^−^, not counting further isotopic signals). Two further lipid ion series ([M+NO_3_]^−^ and [M+Na(OAc)_2_]^−^) were deduced to occur from the same or similar ceramide species. Fifty-eight of these signals displayed significant differences in either direction across the three cell lines (ANOVA, *q* < 0.05). In the LC-MS data set, no additional NO_3_^−^ adducts were observed but instead multiply charged species at low *m/*z values corresponding to the more intense singly charged ions.

Based on the LC-MS data set, the overall mean decrease across ceramides relative to uninfected cells was 62% in Aa23.*w*Mel and 20% in Aa23.*w*MelPop (Fig. 4), although some ceramide species were decreased by as much as 90% in Aa23.*w*Mel, e.g., Cer 41:1 (Lipid Maps match d18:1/23:0) (see Fig. S17 in the supplemental material). Similar patterns emerged in both the DIMS and LC-MS data for Aa23.*w*Mel, as all significantly altered ceramide species decreased (see Fig. S17). Ceramide signals in Aa23.*w*MelPop were inconsistent in their direction of change compared to those in Aa23-T. Changes in two ceramide species, Cer 34:0 and Cer 36:0, were classed as insignificant by the ANOVA but ranked very highly in the LC-MS PLS-DA models and therefore need to be considered. Based on MS/MS data, they were putatively identified as Cer (d14:0/20:0) and Cer (d16:0/20:0) in accordance with the mechanism proposed by Hsu and Turk (see Fig. S13 and S14 in the supplemental material) (54).

Ceramide is often converted to more complex forms, e.g., by glycosylation. Glucosylceramide (GlcCer) is known to be a precursor of much more complex glycophospholipids, although its own role in insects is undefined. In D. melanogaster, it is posited that GlcCer synthesis operates to reduce the ceramide levels and thus lower the apoptosis risk. Other neutral glycosphingolipids containing different monosaccharide headgroups, such as galactosylceramide (GalCer), are primarily found in brain and nervous system tissues in mammals, but little information is available on potential roles in insects. Both lipid classes were detected, but it was here not possible to positively distinguish between GalCer and GlcCer species with the same molecular mass; therefore, they are grouped as monohexosylceramides (HexCers) (Table 1). Among the [M+H]^−^ and [M+OAc]^−^ ions, 16 out of 17 and 10 out of 12, respectively, demonstrated significant changes (ANOVA, *q* < 0.05). In Aa23.*w*Mel, all HexCer species decreased by a mean of 32%, while in Aa23.*w*MelPop, both decreases and increases were observed (average increase of 5%). As for ceramides, the highest increases in Aa23.*w*MelPop were observed for saturated species (HexCer 34:0 and 36:0). Less clear patterns were observed in the remaining species, although they seemed to follow a pattern similar to that of the simple ceramides.

**TABLE 1:** Count of changes in annotated lipid classes in the LC-MS data set^*a*^

Lipid class^*b*^	No. of changes in lipid class (*w*Mel/*w*MelPop)^*c*^				
	−/−	+|+	−/+	+/−	None
Sphingolipids					
Ceramide	42	0	11	0	4
HexCer	15	0	11	0	3
LacCer	4	2	0	3	5
SM	2	1	6	0	3
PE-Cer	14	0	1	0	0
Other phospholipids					
PC	21	5	15	0	9
PE	33	26	0	0	13
PS	2	5	0	0	4
PI	4	21	0	8	19
PG	8	18	5	5	16
Diglycerides	6	4	10	0	10

a Data indicate direction of statistically significant changes in the abundance of lipid classes for Aa23.*w*Mel and Aa23.*w*MelPop, respectively (ANOVA, *q* < 0.05). Counts represent signals, some of which are different ion forms of the same lipid species (singly charged ions only).

b HexCer, monohexosylceramides; LacCer, lactosylceramide; SM, sphingomyelin; PE-Cer, phosphatidylethanolamine ceramide; PC, phosphatidylcholine; PE, phosphatidylethanolamine; PS, phosphatidylserine; PI, phosphatidylinositol; PG, phosphatidylglycerol; DG, diacylglycerol (PC, PE, PS, PI, and PG are diacyl species).

c −/−, downregulated for both *w*Mel and *w*MelPop; +/+, upregulated for both; −/+, downregulated for *w*Mel and upregulated for *w*MelPop; +/−, upregulated for *w*Mel and downregulated for *w*MelPop; none, not significantly changed.

Lactosylceramides (LacCer) were also detected in the LC-MS data set, with 9 of 14 species showing significant alterations in Wolbachia-infected cells. Longer-chain LacCer species were decreased in both Aa23.*w*Mel and Aa23.*w*MelPop; e.g., LacCer 40:1 (annotated by MI-Pack as d18:1/22:0) was reduced by 81% in Aa23.*w*Mel and 51% in Aa23.*w*MelPop. However, species with shorter fatty acid chains (LacCer 34:1 and 36:1) were increased in Aa23.*w*Mel by 1.52- to 1.90-fold and showed limited changes in Aa23.*w*MelPop.

The clear trend across most ceramides was for depletion in the presence of higher-density Wolbachia infections (Fig. 4; see Fig. S17 in the supplemental material). This was also reflected in levels of sphingomyelin (SM), which consists of a ceramide with a phosphorylcholine (PC) headgroup. SM is not synthesized by dipterans (55) but in cell culture could potentially be incorporated from lipoproteins in FBS. Dipterans *de novo* synthesize phosphatidylethanolamine ceramides (PE-Cer), a structural analogue of SM with a phosphatidylethanolamine (PE) instead of a PC headgroup, which functionally replaces SM in insect plasma membranes but imparts different membrane properties (16).

Of several automatically annotated SM [M-H]^−^ and [M+OAc]^−^ signals, the former series could be identified as 15 likely PE-Cer species (see Table S2 in the supplemental material), and it is possible that more signals represent PE-Cer. All were significantly decreased in Aa23.*w*Mel compared to Aa23-T, with the largest decreases in PE-Cer 42:1, 42:2, 40:1, and 39:1. Similarly, 15 of 16 PE-Cer species decreased in Aa23.*w*MelPop, but PE-Cer 34:0 (+2% increase) and another saturated species, PE-Cer 36:0, were among the least changed. This pattern clearly followed that seen in ceramides (see Fig. S17), although in this case, no strongly increasing signals were seen. Of the 10 remaining species annotated as SM, seven were significantly differentially regulated in Wolbachia-infected lines. The mean decrease across sphingomyelin species in Aa23.*w*Mel was 35% (Fig. 4), while Aa23.*w*MelPop saw a 28% increase. All but one species (SM 42:3) were downregulated in the more densely *w*Mel-infected line, especially two saturated species (SM 38:0 and 34:0). However, in Aa23.*w*MelPop, five species were upregulated without a clearly recognizable pattern. There is no obvious correlation with chain size or saturation.

One shared pattern between Cer, HexCer, PE-Cer, and SM was the extreme difference in the change of the 34:0 species, strongly decreased in Aa23.*w*Mel and strongly increased in Aa23.*w*MelPop, making Cer 34:0 the most important species in both the control versus infection model and the Aa23.*w*Mel versus Aa23.*w*MelPop PLS-DA model. The biological relevance of this is unclear.

**FIG 5:**
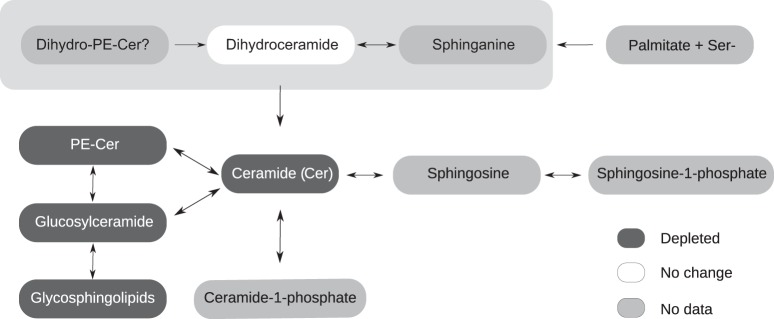
The sphingolipid metabolic network is modulated in Wolbachia-infected A. albopictus Aa23 cell lines. A simplified diagram of sphingolipid metabolism (in accordance with reference 16) is shown. Ceramide is synthesized *de novo* in the endoplasmic reticulum from serine and palmitoyl-coenzyme A through the intermediary metabolites sphinganine and dihydroceramide. Ceramide is then trafficked to the Golgi apparatus, where glycosphingolipids and phosphatidylethanolamine ceramide (PE-Cer) are made and transported to the plasma membrane. In most other eukaryotes, sphingomyelin (SM) is dominant and PE-Cer is a minor lipid, but in general dipterans do not generate SM. Ceramide is broken down into sphingosine and sphingosine-1-phosphate in various intracellular compartments to be recycled or further metabolized. PE-Cer is thought to be hydrolyzed to dihydroceramide as for SM (59). Colors indicate the relative regulation of each lipid class in Aa23.*w*Mel compared to Aa23-T, as derived from LC-MS data. Dark gray, downregulation; white, no change; light gray, no data.

Not all precursors of sphingolipid pathways, such as sphingosine, were detected by either DIMS or LC-MS, but an overall picture of the sphingolipid metabolic network was nevertheless obtained (Fig. 5). For example, 15 of the 17 ceramide signals which showed no significant overall change or showed a small reduction only in the Aa23.*w*Mel line were, according to the Lipid Maps database matches, dihydroceramides, an intermediate in the *de novo* synthesis pathway. There is insufficient information to determine if this is because metabolic flux is concentrated on other pathways and therefore the dihydroceramide pool is being retained or if the dihydroceramide pool is actively utilized as a source of ceramide but an intervening rate-limiting step still results in overall ceramide reduction.

### Diglycerides.

**FIG 6:**
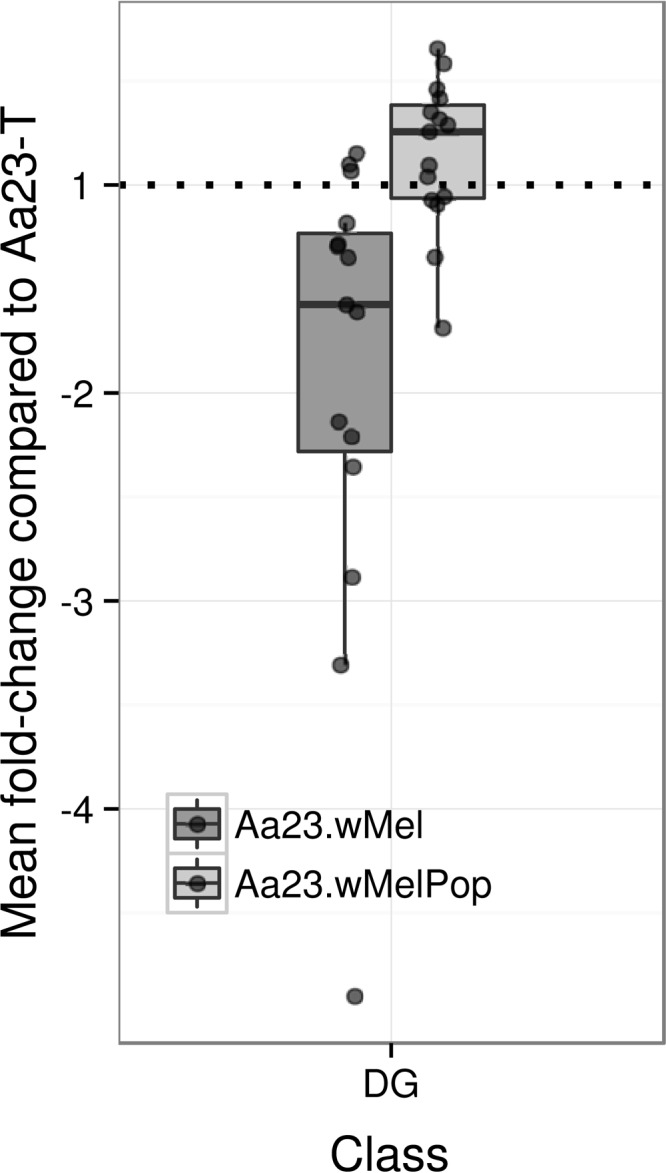
Diacyglycerols (DGs) are modulated in Wolbachia-infected A. albopictus Aa23 cell lines. A box plot shows the mean fold changes (see description in the legend to Fig. 4) in DG species abundance ([M+OAc]^−^ ion series only) in Wolbachia-infected A. albopictus cell lines Aa23.*w*Mel and Aa23.*w*MelPop relative to uninfected Aa23-T. The horizontal dotted line depicts no change.

Few single-chain fatty acids (monoacylglycerols [MGs]) were included in the data sets, so a general analysis is not possible. Diacylglycerols (DGs) were abundant in the LC-MS data set but less so in the DIMS data set. DGs are involved in secondary signaling and are major components of lipid droplets alongside triglycerides (TGs), which were not analyzed here. In the LC-MS data set, 29 DG species remain after the removal of minor ion forms and isotopes, consisting of [M+OAc]^−^ and [M+Cl]^−^ series. Most of these are significantly changed according to the ANOVA (*q* < 0.05), i.e., 15 out of 23 and 5 out of 6, respectively. Both Aa23.*w*Mel and Aa23.*w*MelPop show decreases and increases in DG species abundance compared to Aa23-T across the class (Fig. 6), but with an average decrease of 32% for Aa23.*w*Mel and an average increase of 17% for Aa23.*w*MelPop. The pattern of change by saturation and carbon chain length (see Fig. S18 in the supplemental material) is more similar to that observed for ceramides (see Fig. S17) in Aa23.*w*Mel than in Aa23.*w*MelPop.

Interestingly, in this LC-MS data set, the only lipid in the top 25 signals ranked by variable importance in all the PLS-DA models that was not a sphingolipid or phospholipid was DG(16:0/18:1) ([M+OAc]^−^) or an isomer, which is reduced by 65% in Aa23.*w*Mel but increased by 42% in Aa23.*w*MelPop. The changes of the [M+Cl]^−^ form follow the same directions but are less extreme. Overall, the significance of the changes and distribution is unclear. A previous report correlated increased odd-chain fatty acids with Wolbachia infection (33); the few DG species observed here that clearly contain odd chains (DG 29:1, 31:1, and 31:2) show mostly intensity increases. In addition, the interpretation of the D. melanogaster data was based on its lack of capacity to synthesize odd-chain fatty acids, which would therefore be supplied by symbionts or gut microflora. This interpretation does not hold in cell cultures, as the medium is supplemented with mammalian-derived serum (FBS), which contains odd-chained FAs and therefore will confound or mask a potential effect of Wolbachia infection.

### Other phospholipids.

**FIG 7:**
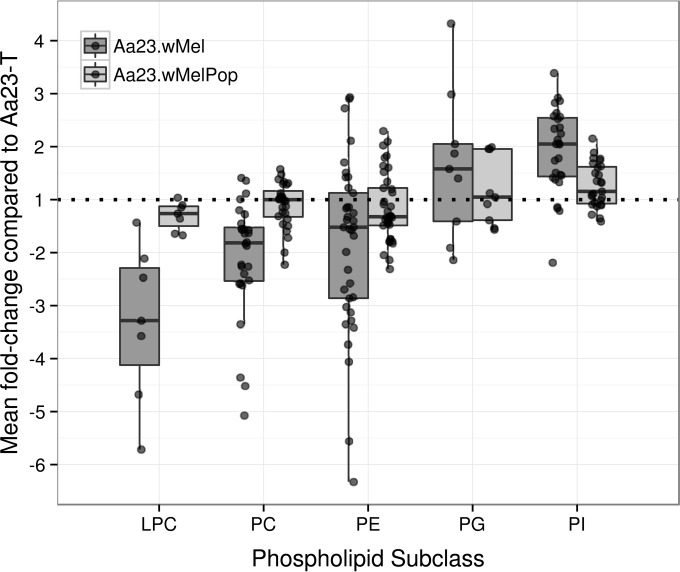
Some phospholipid classes are modulated in Wolbachia-infected A. albopictus Aa23 cell lines. A box plot shows mean fold changes (see description in the legend to Fig. 4) in phospholipid species abundance across selected classes in Aa23.*w*Mel and Aa23.*w*MelPop relative to Aa23-T. The horizontal dotted line depicts no change. LPC, lyso-phosphatidylcholine; PC, phosphatidylcholine (both [M+OAc]^−^ ion series only); PE, phosphatidylethanolamine; PG, phosphatidylglycerol; PI, phosphatidylinositol (all [M-H]^−^ ion series only).

Phospholipids carry out a wide range of functions in the cell; most critically, they are able to form lipid bilayers and are therefore required in the plasma membrane, but many also participate in signaling. Phosphatidylethanolamines (PEs) and phosphatidylcholines (PCs) are the primary components of cellular membranes in D. melanogaster and have previously been found in high abundance in mosquitoes (16). PCs are cylindrically shaped and spontaneously form lipid bilayers, while PEs are cone shaped and thought to help control membrane curvature and to increase membrane fluidity. PCs provide the headgroup for SM and PEs for Cer-PE. Phosphatidylserine (PS) species are thought to be important for recognizing and removing apoptotic cells. A range of differences in abundance was found across the Wolbachia-infected cell lines (Fig. 7).

PS, PCs, and PEs cannot be clearly distinguished by simple mass spectrometry alone, as PE species are isobaric with PC species and acetate adducts of PC species are isobaric with deprotonated PS species. They can be distinguished based on intensity profiles, retention times, and MS/MS profiles, but here we focus on manually curated annotations (see Tables S15 and S16 in the supplemental material) among the 272 species annotated by MI-Pack as PC, PE, PS, or their diacyl- or lysoforms in the LC-MS data set. The DIMS data set contains 136 PC, PE, and PS signals, but there are a number of mixed species and these are more challenging to assign than LC-MS signals, for which isobaric PE and PC species are unlikely to have the same retention time. Similar considerations also hold true for annotations of phosphatidylinositols (PIs) and phosphatidylglycerols (PGs), so the following analysis concentrates on LC-MS data.

Forty-four out of 53 (diacyl) PC signals were identified with significant differential regulation in Wolbachia-infected lines. There was an overall decrease of 38% in Aa23.*w*Mel species and just 4% in Aa23.*w*MelPop species (Fig. 7), but the picture by species was more complicated, with the vast majority showing up to an 80% decrease in Aa23.*w*Mel but seeming inconsistency in Aa23.*w*MelPop (see Fig. S19). In the DIMS data set, only 13 significantly changed [M+OAc]^−^ ions could be annotated with some certainty, but they also showed two clear trends: a decrease of longer-chain PCs and an increase of shorter-chains in the infected cells, which were exaggerated in Aa23.*w*MelPop and Aa23.*w*Mel, respectively.

For lyso-PCs (LPCs), 7 of 11 decrease in Aa23.*w*Mel, i.e., from unsaturated LPC 18:1 and 16:1 by around 80% to saturated LPC 14:0 by 52% and LPC 16:0 by 30%. In Aa23.*w*MelPop, LPC 18:1 and 17:1 show the largest decrease, but LPC 16:0, the least decreased species in Aa23.*w*Mel, is the only increased LPC in both the LC-MS and DIMS data sets. The overall decrease in PC levels parallels the trend for a greater decrease of saturated lipids seen for sphingolipid depletion in Aa23.*w*Mel, while again trends are less clear for Aa23.*w*MelPop.

PS species are important for recognizing and removing apoptotic cells. No PS species was featured in the top 100 signals (ranked by variable importance) for either Wolbachia cell line infection, and most of the 147 species initially designated putative PS by automatic annotation are likely PC adducts. Just 8 of the remaining 12 show a significant change, of which 7 increase in both cell lines.

For lyso-PEs (LPEs), only two of nine were significantly changed: LPE 16:0 increased and LPE 20:0 decreased in both infected cell lines, although LPE 20:1 also decreased significantly according to the DIMS data set. In contrast, 61 of 81 (75%) of diacyl-PEs (PEs) showed differential regulation in the LC-MS data (see Fig. S20 in the supplemental material). Differences between Aa23-T and Aa23.*w*Mel were clear and followed a trend in abundance that was much more conserved across Aa23.*w*Mel and Aa23.*w*MelPop than in the PCs presented above (see Fig. S20). The PE class has the most intense ions in the spectra (see Fig. S21) and includes some multiply charged ion forms, which facilitates their interpretation. Despite the large changes for some species, the average change for both infected cell lines was just a 2% increase (Fig. 7). Only a few multiunsaturated PE species could be rigorously annotated in the DIMS data set. For these limited data, we again see in both cell lines that fold changes depend on molecular weight and there is an overall intensity increase.

Phosphatidylinositol lipids (PIs) induce the PI 3-kinase (PI3K) pathway, which plays an essential role in cell survival and growth. Among 59 PI signals, 30 were significantly changed in the ANOVA (*q* < 0.05), with the largest mean increase of any lipid class considered at 94% in Aa23.*w*Mel and 27% in Aa23.*w*MelPop. Only four of these 30 decreased in Aa23.*w*Mel (PI 44:7, 40:8, 38:4, and 35:2) but with no clear trend in chain length or saturation, although the most reduced of these were also two of the three longest species. Patterns were more visible in data from Aa23.*w*MelPop, where the 11 decreasing signals contained (with the exception of PI 32:2) three to eight double bonds and the 11 most increased species (>40%) contained zero to two double bonds (with the exception of PI 36:5), including all six saturated species.

Phosphatidylglycerols (PG), the lipid class with the second most intense ions in the spectra, were also mostly elevated across the 40 significantly changed signals in this class (mean percent change without multiply charged ions: for Aa23.*w*Mel, 50% increase; for Aa23.*w*MelPop, 17% increase), similar to PIs. As was typical across all lipid classes, changes were usually in the same direction in both Aa23.*w*Mel and *w*MelPop, with larger effect sizes in the more densely infected Aa23.*w*Mel. The eight signals in Aa23.*w*Mel and four in Aa23.*w*MelPop that were >30% lower all featured at least four double bonds, while those with >50% increases contained only one or no double bond, with two exceptions (43:3 and 36:2). It is interesting to note that in these >50% increased samples, wherever MS/MS data are available, the *m/z* 281 ion corresponding to oleic acid is very intense. The largest increases were seen in PG 36:2 and 34:1; these and PG 34:0 are also among the more important “forward selected” species in the corresponding PLS-DA models.

## DISCUSSION

We used DIMS and LC-MS to profile the lipidome of Wolbachia-infected A. albopictus cells and observed a distinct segregation between uninfected and infected cells, which was heightened at a high Wolbachia density. The two data sets were largely consistent in their results. High-density Wolbachia infection resulted in the downregulation of several sphingolipids, which as a lipid class are known to affect membrane structure, fluidity, curvature, and protein complex assembly in lipid rafts (57). Sphingolipids and other identified lipids with significant changes, such as PCs and PIs, also function as signaling molecules and control important pathways, such as trafficking, autophagy, and apoptosis.

DG species showed a variety of responses, which overall indicates minor depletions in DG in the densely infected Aa23.*w*Mel line. It is unclear how this relates to flux in fatty acid synthesis and degradation, because many straight-chain free fatty acids and monoacylglycerols could not be sufficiently annotated (mostly because of a high background signal, for example, due to saturated fatty acids in the solvents). This can be remedied by optimization of extraction procedures and mass spectrometry setup for this lipid class, but no clear inferences can be drawn from the current data. Little evidence has been found elsewhere for a major impact of Wolbachia on fatty acid metabolism; one study found a 2.36-fold increase in fatty acid synthase (FAS) mRNA expression in *w*Mel-infected A. aegypti mosquitoes (15).

While other lipids were modulated and each class included at least a few species with quite noteworthy differences, the class changes in sphingolipids, PC, and PI are significant and immediately suitable for the design of downstream functional assays. In combination, the data observed suggest a substantial reprogramming of the host lipid metabolic network. However, the flux within the system and the mechanism of this modulation have yet to be elucidated. Our observations are contextualized below in relation to the current knowledge about lipids and their interactions with similar bacteria, viruses, and hosts.

### Wolbachia-host interactions and lipid metabolism.

Wolbachia *w*Mel has a highly reduced genome with limited lipid metabolic capabilities (27). It only retains genes for fatty acid elongation and limited transformations of glycerolipids and glycerophospholipids. While many other obligate intracellular bacteria have the capacity to degrade but not produce sphingolipids, Wolbachia bacteria have neither of these gene pathways (27). This means that changes in lipid composition are unlikely to be due to the direct action of Wolbachia metabolic enzymes but are more likely to involve a host response to Wolbachia infection or manipulation of host lipid pathways by Wolbachia.

The changes observed in our analysis might have implications for Wolbachia maintenance and infectivity through alteration of structural membrane properties. Specific depletion of bioactive sphingolipids such as Cer and PE-Cer was observed in Wolbachia-infected A. albopictus mosquitoes. This finding is interesting when considered with previous evidence for cholesterol depletion in Wolbachia-infected mosquitoes (29) because, in contrast, sphingolipids increase in cholesterol-depleted D. melanogaster and are hypothesized to play an analogous and compensating structural role (58). Our lipidomics analyses revealed no findings on sterols, but future work combining sterol data with functional testing of membrane properties during Wolbachia infection could be revealing.

Sphingomyelin-, cholesterol-, and ceramide-depleted membranes are more fluid than enriched membranes (58, 59), and this could be advantageous for Wolbachia bacteria, as cellular membranes are reconfigured to provide the double membrane-bound, Golgi apparatus-derived intracellular vesicles in which they dwell (60). However, these lipids are also involved in forming lipid rafts in plasma membranes which most evidence suggests are useful for bacterial invasion and persistence (61). One possibility to explore is that sphingolipid depletion limits autophagy and apoptosis even where Wolbachia is causing cellular stress. This hypothesis fits with our current knowledge that activation of autophagy can reduce Wolbachia bacterial density (30) and that accumulated ceramides are able to activate both autophagic and apoptotic pathways by interacting with the mTOR pathway (62, 63) and have been shown to target autophagosomes to mitochondria (63).

Phospholipid classes showed more moderate overall changes, although within each class there were clearly differentially regulated species. The clearest modulation in phospholipids was a 2-fold increase in PIs in Aa23.*w*Mel and an overall downregulation of PCs. Due to a paucity of information on the role of specific phospholipid species in insects, the functional significance of these findings is difficult to predict. PIs play key roles in receptor-mediated signal transduction, membrane dynamics, and cell remodelling via actin and other cytoskeletal components (64). Several vacuolar pathogens are known to modulate PI levels either directly through production of PI metabolic enzymes or through manipulation of host pathways (65, 66). The Wolbachia genome does not encode PI-modifying enzymes, but it could manipulate or subvert host pathways and there is a precedent for a PI increase in bacterial infection. For example, Shigella flexneri upregulates various PI species (67) postentry, and this is thought to provide protection from apoptosis. PIs thus provide another likely candidate lipid class for involvement in the maintenance and regulation of Wolbachia infections.

PCs are primarily structural molecules and also play a role in the aforementioned lipid raft domains. They are expected to form a major part of intracellular bacterial membranes, but the composition of the Wolbachia membrane is currently unknown. Many bacterial infections appear to increase rather than deplete PCs, and several pathogenic bacteria are able to synthesize their own using host-provided choline (68), which Wolbachia could not as it lacks the necessary genes. The mechanism by which abundance is altered and any functional impacts on the Wolbachia-host relationship thus remain to be established, and there are few predictions that can be inferred from other systems in this case.

### Implications for pathogen transmission.

Host lipids are vital to the viral and bacterial life cycle; therefore, it might be expected that both Wolbachia bacteria and DENV would evolve the means to modulate the cellular lipidome to ensure their own preservation and replication or the means to thrive in spite of lipid-mediated host responses to infection. This expectation appears to be upheld in DENV-infected A. albopictus C6/36 cells, in which the cellular lipid profile was substantially altered upon infection in a way that favors viral propagation (16).

The data presented here are striking in comparison to observations on DENV infection, because the same lipid classes that are depleted in Wolbachia-infected A. albopictus Aa23 cells are selectively enriched in DENV-infected A. albopictus C6/36 cells (16). Sphingomyelin and ceramides were upregulated in both the whole cell and the replication complex membranes during DENV infection, including enrichment of specific ceramide species in the 16,000-molecular-weight pellet containing the replication complex. This may be a cellular response or might reflect a direct need for ceramides in the viral life cycle (16), particularly in forming negative curvature of lipid bilayers as observed in some DENV-induced membrane structures. Sphingolipids have also been shown to be important in the pathogenesis of a variety of other intracellular pathogens, including Plasmodium parasites, regulating host-microbe interactions (69).

In addition to generating favorable membrane properties, ceramide enrichment in DENV infection also appears to upregulate autophagy for fatty acid provision, and this is a requirement for viral replication in both mammalian and mosquito cells (16, 19, 70). Ceramide also increases apoptosis in some circumstances, but DENV infection antagonizes apoptosis pathways and is a persistent infection, so it seems unlikely that ceramide is being utilized for this purpose. As Wolbachia bacteria are potentially targeted by autophagy (30) and are therefore under significant selection pressure to subvert or downregulate the process, Wolbachia and DENV oppose each other in terms of host cell lipid regulation, which could be significant with respect to virus transmission blocking. Likewise, unsaturated PC, depleted in the presence of Wolbachia, was the most increased phospholipid in DENV-infected cells, perhaps due to its role in membrane curvature and maintaining fluid membranes (16), potentially aiding remodelling by viruses.

The results presented have provided new directions for research into Wolbachia-induced modulation of host lipid metabolism and implications for DENV infection and transmission. Some caution is needed in interpreting results, since the cell line used was derived from A. albopictus eggs, and further studies should examine different tissues in adult insects, such as the fat body, and in organs critical to virus transmission, i.e., midguts and salivary glands. This will be particularly informative given that although the Aa23 cell line is heterogeneous (40), its composition will not fully reflect cell differentiation in adult mosquitoes, in which certain cells such as adipocytes are known to store more fat than others. Whole-organism studies are also necessary to ascertain the lipid environments likely to be encountered by pathogens and Wolbachia through their natural life cycle. Lipid profiles can differ at the tissue level; for instance, the fat body is know to play a specific role in lipid storage and metabolism, meaning that both the lipid environment and distribution of microorganisms in different tissues will alter during development and the course of infections. Diet, age, and development can also be controlled in whole mosquitoes, while cell line growth and proliferation can be variable, all of which may impact lipid profiles.

Due to the different direction and not simply magnitude of some lipid alterations observed at increasing Wolbachia density in the Aa23 lines, the role of density and the relative impact of Wolbachia-induced changes in host lipid metabolism in comparison to the role of Wolbachia metabolism itself need to be examined. Subcellular investigation will also be informative; for example, different lipid profiles have been found in both the endoplasmic reticulum and 16,000-molecular-weight replication complex during DENV infection (16). A more detailed analysis of both lipid-bound and free fatty acids and various sterols and derivatives would be also be very useful, because this investigation did not provide sufficient data on these classes of lipids and related metabolites, which have already been linked to Wolbachia-host interactions (29). The multiple effects of Wolbachia on the cellular lipid profile mean that determining which components directly affect viral replication will not be straightforward, since it seems likely that modulation of multiple lipid species will contribute to viral inhibition. The role of sphingolipids is a priority area for future research and should add to an emerging picture of the molecular mechanisms underpinning Wolbachia-mosquito-virus interactions.

## Supplementary Material

Supplemental material

## References

[1] Hoffmann AA, Turelli M 1997 Cytoplasmic incompatibility in insects, p 42–80. *In* O'Neill S, Hoffmann AA, Werren J (ed), Influential passengers: microorganisms and invertebrate reproduction. Oxford University Press, Oxford, United Kingdom.

[2] Kambris Z, Cook PE, Phuc HK, Sinkins SP 2009 Immune activation by life-shortening Wolbachia and reduced filarial competence in mosquitoes. Science 326:134–136. doi:10.1126/science.1177531. 19797660 PMC2867033

[3] Moreira LA, Iturbe-Ormaetxe I, Jeffery JA, Lu G, Pyke AT, Hedges LM, Rocha BM, Hall-Mendelin SS, Day A, Riegler M, Hugo LE, Johnson KN, Kay BH, McGraw EA, van den Hurk AF, Ryan PA, O'Neill SL 2009 A Wolbachia symbiont in Aedes aegypti limits infection with dengue, chikungunya, and Plasmodium. Cell 139:1268–1278. doi:10.1016/j.cell.2009.11.042. 20064373

[4] Kambris Z, Blagborough AM, Pinto SB, Blagrove MS, Godfray HC, Sinden RE, Sinkins SP 2010 Wolbachia stimulates immune gene expression and inhibits Plasmodium development in Anopheles gambiae. PLoS Pathog 6:e1001143. doi:10.1371/journal.ppat.1001143. 20949079 PMC2951381

[5] Bian G, Xu Y, Lu P, Xie Y, Xi Z 2010 The endosymbiotic bacterium Wolbachia induces resistance to dengue virus in Aedes aegypti. PLoS Pathog 6:e1000833. doi:10.1371/journal.ppat.1000833. 20368968 PMC2848556

[6] Walker T, Johnson PH, Moreira LA, Iturbe-Ormaetxe I, Frentiu FD, McMeniman CJ, Leong YS, Dong Y, Axford J, Kriesner P, Lloyd AL, Ritchie SA, O'Neill SL, Hoffmann AA 2011 The *w*Mel Wolbachia strain blocks dengue and invades caged Aedes aegypti populations. Nature 476:450–453. doi:10.1038/nature10355. 21866159

[7] Blagrove MSC, Arias-Goeta CA, Failloux A, Sinkins SP 2012 The Wolbachia strain *w*Mel induces cytoplasmic incompatibility in and blocks dengue transmission by Aedes albopictus. Proc Natl Acad Sci U S A 109:255–260. doi:10.1073/pnas.1112021108. 22123944 PMC3252941

[8] Blagrove MSC, Arias-Goeta CA, Di Genua C, Failloux A-B, Sinkins SP 2013 Wolbachia wMel transinfection in Aedes albopictus is not detrimental to host fitness and inhibits chikungunya virus. PLoS Negl Trop Dis 7:e2152. doi:10.1371/journal.pntd.0002152. 23556030 PMC3610642

[9] Bian G, Joshi D, Dong Y, Lu P, Zhou G, Pan X, Xu Y, Dimopoulos G, Xi Z 2013 Wolbachia invades Anopheles stephensi populations and induces refractoriness to Plasmodium infection. Science 340:748–751. doi:10.1126/science.1236192. 23661760

[10] van den Hurk AF, Hall-Mendelin S, Pyke AT, Frentiu FD, McElroy K, Day A, Higgs S, O'Neill SL 2012 Impact of Wolbachia on infection with chikungunya and yellow fever viruses in the mosquito vector Aedes aegypti. PLoS Negl Trop Dis 6:e1892. doi:10.1371/journal.pntd.0001892. 23133693 PMC3486898

[11] Hoffmann AA, Montgomery BL, Popovici J, Iturbe-Ormaetxe I, Johnson PH, Muzzi F, Greenfield M, Durkan M, Leong YS, Dong Y, Cook H, Axford J, ACallahan AG, Kenny N, Omodei C, McGraw EA, Ryan PA, Ritchie SA, Turelli M, O'Neill SL 2011 Successful establishment of Wolbachia in Aedes populations to suppress dengue transmission. Nature 476:454–457. doi:10.1038/nature10356. 21866160

[12] Hoffmann AA, Iturbe-Ormaetxe I, Callahan AG, Phillips BL, Billington K, Axford JK, Montgomery B, Turley AP, O'Neill SL 2014 Stability of the *w*Mel Wolbachia infection following invasion into Aedes aegypti populations. PLoS Negl Trop Dis 8:e3115. doi:10.1371/journal.pntd.0003115. 25211492 PMC4161343

[13] Frentiu FD, Zakir T, Walker T, Popovici J, Pyke AT, van den Hurk A, McGraw EA, O'Neill SL 2014 Limited dengue virus replication in field-collected Aedes aegypti mosquitoes infected with Wolbachia. PLoS Negl Trop Dis 8:e2688. doi:10.1371/journal.pntd.0002688. 24587459 PMC3930499

[14] Pan X, Zhou G, Wu J, Bian G, Lu P, Raikhel AS, Xi Z 2012 Wolbachia induces reactive oxygen species (ROS)-dependent activation of the Toll pathway to control dengue virus in the mosquito Aedes aegypti. Proc Natl Acad Sci U S A 109:E23-31. doi:10.1073/pnas.1116932108. 22123956 PMC3252928

[15] Rancès E, Ye YH, Woolfit M, McGraw EA, O'Neill SL 2012 The relative importance of innate immune priming in Wolbachia-mediated dengue interference. PLoS Pathog 8:e1002548. doi:10.1371/journal.ppat.1002548. 22383881 PMC3285598

[16] Perera R, Riley C, Isaac G, Hopf-Jannasch AS, Moore RJ, Weitz KW, Pasa-Tolic L, Metz TO, Adamec J, Kuhn RJ 2012 Dengue virus infection perturbs lipid homeostasis in infected mosquito cells. PLoS Pathog 8:e1002584. doi:10.1371/journal.ppat.1002584. 22457619 PMC3310792

[17] Carro AC, Damonte EB 2013 Requirement of cholesterol in the viral envelope for dengue virus infection. Virus Res 174:78–87. doi:10.1016/j.virusres.2013.03.005. 23517753

[18] Rothwell C, LeBreton A, Ng CY, Lim JY, Liu W, Vasudevan S, Labow M, Gu F, Gaither LA 2009 Cholesterol biosynthesis modulation regulates dengue viral replication. Virology 389:8–19. doi:10.1016/j.virol.2009.03.025. 19419745

[19] Heaton NS, Randall G 2010 Dengue virus-induced autophagy regulates lipid metabolism. Cell Host Microbe 8:422–432. doi:10.1016/j.chom.2010.10.006. 21075353 PMC3026642

[20] Heaton NS, Randall G 2011 Dengue virus and autophagy. Viruses 3:1332–1341. doi:10.3390/v3081332. 21994782 PMC3185800

[21] Judith D, Mostowy S, Bourai M, Gangneux N, Lelek M, Lucas-Hourani M, Cayet N, Jacob Y, Prévost MC, Pierre P, Tangy F, Zimmer C, Vidalain PO, Couderc T, Lecuit M 2013 Species-specific impact of the autophagy machinery on Chikungunya virus infection. EMBO Rep 14:534–544. doi:10.1038/embor.2013.51. 23619093 PMC3674439

[22] Zaitseva E, Yang S-T, Melikov K, Pourmal S, Chernomordik LV 2010 Dengue virus ensures its fusion in late endosomes using compartment-specific lipids. PLoS Pathog 6:e1001131. doi:10.1371/journal.ppat.1001131. 20949067 PMC2951369

[23] Gay B, Bernard E, Solignat M, Chazal N, Devaux C, Briant L 2012 pH-dependent entry of chikungunya virus into Aedes albopictus cells. Infect Genet Evol 12:1275–1281. doi:10.1016/j.meegid.2012.02.003. 22386853

[24] Poh MK, Shui G, Xie X, Shi P-Y, Wenk MR, Gu F 2012 U18666A, an intra-cellular cholesterol transport inhibitor, inhibits dengue virus entry and replication. Antiviral Res 93:191–198. doi:10.1016/j.antiviral.2011.11.014. 22146564

[25] Shi J, Luo H 2012 Interplay between the cellular autophagy machinery and positive-stranded RNA viruses. Acta Biochim Biophys Sin 44:375–384. doi:10.1093/abbs/gms010. 22343377 PMC7110239

[26] Marquardt MT, Phalen T, Kielian M 1993 Cholesterol is required in the exit pathway of Semliki Forest virus. J Cell Biol 123:57–65. doi:10.1083/jcb.123.1.57. 8408205 PMC2119816

[27] Wu M, Sun LV, Vamathevan J, Riegler M, Deboy R, Brownlie JC, McGraw EA, Martin W, Esser C, Ahmadinejad N, Wiegand C, Madupu R, Beanan MJ, Brinkac LM, Daugherty SC, Durkin AS, Kolonay JF, Nelson WC, Mohamoud Y, Lee P, Berry K, Young MB, Utterback T, Weidman J, Nierman WC, Paulsen IT, Nelson KE, Tettelin H, O'Neill SL, Eisen JA 2004 Phylogenomics of the reproductive parasite Wolbachia pipientis wMel: a streamlined genome overrun by mobile genetic elements. PLoS Biol 2:E69. doi:10.1371/journal.pbio.0020069. 15024419 PMC368164

[28] Hobson RP 1935 On a fat-soluble growth factor required by blow-fly larvae: identity of the growth factor with cholesterol. Biochem J 29:2023. doi:10.1042/bj0292023. 16745871 PMC1266719

[29] Caragata EP, Rances E, Hedges LM, Gofton AW, Johnson KN, O'Neill SL, McGraw EA 2013 Dietary cholesterol modulates pathogen blocking by Wolbachia. PLoS Pathog 9:e1003459. doi:10.1371/journal.ppat.1003459. 23825950 PMC3694857

[30] Voronin D, Cook DA, Steven A, Taylor MJ 2012 Autophagy regulates Wolbachia populations across diverse symbiotic associations. Proc Natl Acad Sci U S A 109:E1638–E1646. doi:10.1073/pnas.1203519109. 22645363 PMC3382551

[31] Teixeira L, Ferreira A, Ashburner M 2008 The bacterial symbiont Wolbachia induces resistance to RNA viral infections in Drosophila melanogaster. PLoS Biol 6:E2. doi:10.1371/journal.pbio.1000002. 19222304 PMC2605931

[32] Kudchodkar SB, Levine B 2009 Viruses and autophagy. Rev Med Virol 19:359–378. doi:10.1002/rmv.630. 19750559 PMC2852112

[33] Scheitz CJ, Guo Y, Early AM, Harshman LG, Clark AG 2013 Heritability and inter-population differences in lipid profiles of Drosophila melanogaster. PLoS One 8:e72726. doi:10.1371/journal.pone.0072726. 24013349 PMC3754969

[34] Bonizzoni M, Gasperi G, Chen X, James AA 2013 The invasive mosquito species Aedes albopictus: current knowledge and future perspectives. Trends Parasitol 29:460–468. doi:10.1016/j.pt.2013.07.003. 23916878 PMC3777778

[35] Bhatt S, Gething PW, Brady OJ, Messina JP, Farlow AW, Moyes CL, Drake JM, Brownstein JS, Hoen AG, Sankoh O, Myers MF, George DB, Jaenisch T, Wint GR, Simmons CP, Scott TW, Farrar JJ, Hay SI 2013 The global distribution and burden of dengue. Nature 496:504–507. doi:10.1038/nature12060. 23563266 PMC3651993

[36] Burt FJ, Rolph MS, Rulli NE, Mahalingam S, Heise MT 2012 Chikungunya: a re-emerging virus. Lancet 379:662–671. doi:10.1016/S0140-6736(11)60281-X. 22100854

[37] Sommer U, Herscovitz H, Welty FK, Costello CE 2006 LC-MS-based method for the qualitative and quantitative analysis of complex lipid mixtures. J Lipid Res 47:804–814. doi:10.1194/jlr.M500506-JLR200. 16443931

[38] Southam AD, Payne TG, Cooper HJ, Arvanitis TN, Viant MR 2007 Dynamic range and mass accuracy of wide-scan direct infusion nanoelectrospray Fourier transform ion cyclotron resonance mass spectrometry-based metabolomics increased by the spectral stitching method. Anal Chem 79:4595–4602. doi:10.1021/ac062446p. 17511421

[39] Viant MR, Sommer U 2013 Mass spectrometry based environmental metabolomics: a primer and review. Metabolomics 9:S144–S158.

[40] O'Neill SL, Pettigrew M, Sinkins SP, Braig HR, Andreadis TG, Tesh RB 1997 *In vitro* cultivation of Wolbachia pipientis in an Aedes albopictus cell line. Insect Mol Biol 6:33–39. doi:10.1046/j.1365-2583.1997.00157.x. 9013253

[41] Molloy JC, Sinkins SP 2015 Wolbachia do not induce reactive oxygen species-dependent immune pathway activation in Aedes albopictus. Viruses 7:4624–4639. doi:10.3390/v7082836. 26287231 PMC4576197

[42] Glazov EA, Pheasant M, McGraw EA, Bejerano G, Mattick JS 2005 Ultraconserved elements in insect genomes: a highly conserved intronic sequence implicated in the control of homothorax mRNA splicing. Genome Res 15:800–808. doi:10.1101/gr.3545105. 15899965 PMC1142470

[43] Weber RJ, Southam AD, Sommer U, Viant MR 2011 Characterization of isotopic abundance measurements in high resolution FT-ICR and Orbitrap mass spectra for improved confidence of metabolite identification. Anal Chem 83:3737–3743. doi:10.1021/ac2001803. 21466230

[44] Kirwan JA, Weber RJM, Broadhurst DI, Viant MR 2014 Direct infusion mass spectrometry metabolomics dataset: a benchmark for data processing and quality control. Sci Data 1:140012. doi:10.1038/sdata.2014.12. 25977770 PMC4381748

[45] Tautenhahn R, Boettcher C, Neumann S 2008 Highly sensitive feature detection for high resolution LC/MS. BMC Bioinformatics 9:504. doi:10.1186/1471-2105-9-504. 19040729 PMC2639432

[46] Dunn WB, Broadhurst D, Brown M, Baker PN, Redman CW, Kenny LC, Kell DB 2008 Metabolic profiling of serum using ultra performance liquid chromatography and the LTQ-Orbitrap mass spectrometry system. J Chromatogr B 871:288–298. doi:10.1016/j.jchromb.2008.03.021. 18420470

[47] Benjamini Y, Hochberg Y 1995 Controlling the false discovery rate—a practical and powerful approach to multiple testing. J R Stat Soc Series B Stat Methodol 57:289–300.

[48] Weber RJ, Viant MR 2010 MI-Pack: increased confidence of metabolite identification in mass spectra by integrating accurate masses and metabolic pathways. Chem Intel Lab Syst 104:75–82. doi:10.1016/j.chemolab.2010.04.010.

[49] Kanehisa M, Goto S, Sato Y, Kawashima M, Furumichi M, Tanabe M 2014 Data, information, knowledge and principle: back to metabolism in KEGG. Nucleic Acids Res 42:D199–D205. doi:10.1093/nar/gkt1076. 24214961 PMC3965122

[50] Fahy E, Sud M, Cotter D, Subramaniam S 2007 LIPID MAPS online tools for lipid research. Nucleic Acids Res 35:W606–W612. doi:10.1093/nar/gkm324. 17584797 PMC1933166

[51] Fahy E, Subramaniam S, Murphy RC, Nishijima M, Raetz CR, Shimizu T, Spener F, van Meer G, Wakelam MJ, Dennis EA 2009 Update of the LIPID MAPS comprehensive classification system for lipids. J Lipid Res 50:S9–S14. doi:10.1194/jlr.R800095-JLR200. 19098281 PMC2674711

[52] Khoo C, Venard C, Fu Y, Mercer D, Dobson S 2013 Infection, growth and maintenance of Wolbachia pipientis in clonal and non-clonal Aedes albopictus cell cultures. Bull Entomol Res 103:251–260. doi:10.1017/S0007485312000648. 23113940

[53] Lu P, Bian G, Pan X, Xi Z 2012 Wolbachia induces density-dependent inhibition to dengue virus in mosquito cells. PLoS Negl Trop Dis 6:e1754. doi:10.1371/journal.pntd.0001754. 22848774 PMC3404113

[54] Hsu F-F, Turk J 2002 Characterization of ceramides by low energy collisional-activated dissociation tandem mass spectrometry with negative-ion electrospray ionization. J Am Soc Mass Spectrom 13:558–570. doi:10.1016/S1044-0305(02)00358-6. 12019979

[55] Rietveld A, Neutz S, Simons K, Eaton S 1999 Association of sterol-and glycosylphosphatidylinositol-linked proteins with Drosophila raft lipid microdomains. J Biol Chem 274:12049–12054. doi:10.1074/jbc.274.17.12049. 10207028

[56] Zheng W, Kollmeyer J, Symolon H, Momin A, Munter E, Wang E, Kelly S, Allegood JC, Liu Y, Peng Q, Ramaraju H, Sullards MC, Cabot M, Merrill AH 2006 Ceramides and other bioactive sphingolipid backbones in health and disease: lipidomic analysis, metabolism and roles in membrane structure, dynamics, signaling and autophagy. BBA Biomembranes 1758:864–1884. 10.1016/j.bbamem.2006.08.009 17052686

[57] van Meer G, Voelker DR, Feigenson GW 2008 Membrane lipids: where they are and how they behave. Nat Rev Mol Cell Biol 9:112–124. doi:10.1038/nrm2330. 18216768 PMC2642958

[58] Carvalho M, Schwudke D, Sampaio JL, Palm W, Riezman I, Dey G, Gupta GD, Mayor S, Riezman H, Shevchenko A, Kurzchalia TV, Eaton S 2010 Survival strategies of a sterol auxotroph. Development 137:3675–3685. doi:10.1242/dev.044560. 20940226 PMC2964098

[59] Hannich JT, Umebayashi K, Riezman H 2011 Distribution and functions of sterols and sphingolipids. Cold Spring Harb Perspect Biol 3:a004762. doi:10.1101/cshperspect.a004762. 21454248 PMC3101845

[60] Cho K-O, Kim G-W, Lee O-K 2011 Wolbachia bacteria reside in host Golgi-related vesicles whose position is regulated by polarity proteins. PLoS One 6:e22703. doi:10.1371/journal.pone.0022703. 21829485 PMC3145749

[61] Lafont F, Van Der Goot FG 2005 Bacterial invasion via lipid rafts. Cell Microbiol 7:613–620. doi:10.1111/j.1462-5822.2005.00515.x. 15839890

[62] Jiang W, Ogretmen B 2014 Autophagy paradox and ceramide. Biochim Biophys Acta 1841:783–792. doi:10.1016/j.bbalip.2013.09.005. 24055889 PMC3960371

[63] Sentelle RD, Senkal CE, Jiang W, Ponnusamy S, Gencer S, Selvam SP, Ramshesh VK, Peterson YK, Lemasters JJ, Szulc ZM, Bielawski J, Ogretmen B 2012 Ceramide targets autophagosomes to mitochondria and induces lethal mitophagy. Nat Chem Biol 8:831–838. doi:10.1038/nchembio.1059. 22922758 PMC3689583

[64] Di Paolo G, De Camilli P 2006 Phosphoinositides in cell regulation and membrane dynamics. Nature 443:651–657. doi:10.1038/nature05185. 17035995

[65] Hilbi H 2006 Modulation of phosphoinositide metabolism by pathogenic bacteria. Cell Microbiol 8:1697–1706. doi:10.1111/j.1462-5822.2006.00793.x. 16939534

[66] Weber SS, Ragaz C, Hilbi H 2009 Pathogen trafficking pathways and host phosphoinositide metabolism. Mol Microbiol 71:1341–1352. doi:10.1111/j.1365-2958.2009.06608.x. 19208094

[67] Pendaries C, Tronchere H, Arbibe L, Mounier J, Gozani O, Cantley L, Fry MJ, Gaits-Iacovoni F, Sansonetti PJ, Payrastre B 2006 PtdIns(5)P activates the host cell PI3-kinase/Akt pathway during Shigella flexneri infection. EMBO J 25:1024–1034. doi:10.1038/sj.emboj.7601001. 16482216 PMC1409730

[68] Geiger O, Lopez-Lara IM, Sohlenkamp C 2013 Phosphatidylcholine biosynthesis and function in bacteria. Biochim Biophys Acta 1831:503–513. doi:10.1016/j.bbalip.2012.08.009. 22922101

[69] Heung LJ, Luberto C, Del Poeta M 2006 Role of sphingolipids in microbial pathogenesis. Infect Immun 74:28–39. doi:10.1128/IAI.74.1.28-39.2006. 16368954 PMC1346627

[70] Heaton NS, Randall G 2011 Multifaceted roles for lipids in viral infection. Trends Microbiol 19:368–375. doi:10.1016/j.tim.2011.03.007. 21530270 PMC3130080

