# Supplementary material for: Wolbachia Modulates Lipid Metabolism in Aedes albopictus Mosquito Cells
Source: Appl Environ Microbiol. 2016 May 2;82(10):3109–20. doi: 10.1128/AEM.00275-16 (PMC4959074; doi:10.1128/AEM.00275-16)
Supplement: Supplemental material [file supp_82_10_3109__index.html]

Supplemental material 

# Wolbachia Modulates Lipid Metabolism in Aedes albopictus Mosquito Cells

## Supplemental material

**Files in this Data Supplement:**

- Supplemental file 1 -

  PLS-?DA supervised model of all three groups (DIMS), with all 2,044 *m/z* values included, built using 4 latent variables and tested using 1,000 permutations (Table S1a); optimal PLS-?DA supervised model (DIMS), comprising 70 forward selected *m/z* values only, built using 4 latent variables and tested using 1,000 permutations (Table S1b); PLS-?DA supervised model of all three groups (LCMS), with all 4,736 *m/z* values included, built using 5 latent variables and tested using 1,000 permutations (Table S2a); optimal PLS-DA supervised model (LCMS), comprising 500 forward selected *m/z* values only, built using 5 latent variables and tested using 1,000 permutations (Table S2b); PLS-?DA supervised model of Aa23-?T (control) versus infected (reclassified: Aa23.wMel + Aa23.wMel Pop) (DIMS), with all 2,044 *m/z* values included, built using 5 latent variables and tested using 1,000 permutations (Table S3a); optimal PLS-?DA supervised model (DIMS), comprising 77 forward selected *m/z* values only, built using 5 latent variables and tested using 1,000 permutations (Table S3b); PLS-?DA supervised model of Aa23-?T (control) versus Aa23.wMel (DIMS), with all 2,044 *m/z* values included, built using 2 latent variables and tested using 1,000 permutations (Table S4a); optimal PLS-?DA supervised model (DIMS), comprising 23 forward selected *m/z* values only, built using 2 latent variables and tested using 1,000 permutations (Table S4b); PLS-?DA supervised model of Aa23-T versus Aa23.wMelPop (DIMS), with all 2,044 *m/z* values included, built using 3 latent variables and tested using 1,000 permutations (Table S5a); optimal PLS-?DA supervised model (DIMS), comprising of 69 forward selected *m/z* values only, built using 3 latent variables and tested using 1,000 permutations (Table S5b); PLS-?DA supervised model of Aa23.wMel versus Aa23.wMelPop (DIMS), with all 2,044 *m/z* values included, built using 2 latent variables and tested using 1,000 permutations (Table S6a); optimal PLS-?DA supervised model (DIMS), comprising 73 forward selected *m/z* values only, built using 2 latent variables and tested using 1,000 permutations (Table S6b); PLS-?DA supervised model of Aa23-?T (control) versus infected (reclassified: Aa23.wMel + Aa23.wMel Pop) (LCMS), with all 4,736 *m/z* values included, built using 2 latent variables and tested using 1,000 permutations (Table S7a); optimal PLS-?DA supervised model (LCMS), comprising 463 forward selected *m/z* values only, built using 2 latent variables and tested using 1,000 permutations (Table S7b); PLS-?DA supervised model of Aa23-?T and Aa23.wMel (LCMS), with all 4,736 *m/z* values included, built using 1 latent variable and tested using 1,000 permutations (Table S8a); optimal PLS-?DA supervised model (LCMS), comprising 59 forward selected *m/z* values only, built using 1 latent variable and tested using 1,000 permutations (Table S8b); PLS-?DA supervised model of Aa23-?T versus Aa23.wMelPop (LCMS), with all 4,736 *m/z* values included, built using 3 latent variables and tested using 1,000 permutations (Table S9a); optimal PLS-?DA supervised model (LCMS), comprising 235 forward selected *m/z* values only, built using 3 latent variables and tested using 1,000 permutations (Table S9b); PLS-?DA supervised model of Aa23.wMel versus Aa23.wMelPop (LCMS), with all 4,736 *m/z* values included, built using 1 latent variable and tested using 1,000 permutations (Table S10a); optimal PLS-?DA supervised model (LCMS), comprising 57 forward selected *m/z* values only, built using 1 latent variable and tested using 1,000 permutations (Table S10b); LC-?MS gradient (Table S11); XCMS/CAMERA script (in R) for processing cdf files (Table S12); MS/MS spectrum of the ceramide signal at *m/z* 538 (Fig. S13); MS/MS spectrum of the ceramide signal at *m/z* 536 (Fig. S14); multiple ceramide species show strong decreases in *Wolbachia*-?infected *Ae. albopictus* Aa23 cell lines (Fig. S17); diacyglycerol species in *Wolbachia*-?infected *Ae. albopictus* Aa23 cell lines show both intensity decreases and increases (Fig. S18); phosphatidylcholine species in *Wolbachia*-infected *Ae. albopictus* Aa23 cell lines show both intensity decreases and increases (Table S19); saturated phosphatidylethanolamine species increase and less saturated species decrease in *Wolbachia*-?infected *Ae. albopictus* Ae23 cell lines (Table S20); phosphatidylethanolamine and phosphatidylcholine lipid signals show highest intensity in LCMS analysis of *Ae. albopictus* Aa23-?T cell lines (Table S21).

  PDF, 845K
- Supplemental file 2 -

  Statistics and MS signal annotations for the DIMS experiments (Table S15)

  XLSX, 3.7M
- Supplemental file 3 -

  Statistics and MS signal annotations for the LC-MS experiments (Table S16)

  XLSX, 5.3M
