# Supplementary material for: Wolbachia Modulates Lipid Metabolism in Aedes albopictus Mosquito Cells
Source: Appl Environ Microbiol. 2016 May 2;82(10):3109–20. doi: 10.1128/AEM.00275-16 (PMC4959074; doi:10.1128/AEM.00275-16)
Supplement: Supplemental material [file AEM.00275-16_zam999117147so1.pdf]

Supplementary information

Jennifer C. Molloy, Ulf Sommer, Mark R. Viant and Steven P. Sinkins:

Wolbachia modulation of lipid metabolism in *Aedes albopictus* mosquito cells

Tables S1-10 & S13-14, figures S1-2 and S17-21.

Tables S1-10: Test results for PLS-DA models (internal cross validation) and forward selection

Table S1a: PLS-DA supervised model of all three groups (DIMS), with all 2044 *m/z* values included, built using 4 latent variables and tested using 1000 permutations. These values indicate the validity of the model, highlighting the metabolic differences between the three classes.

class	class error rate	p value
Aa23-T	0.1505	0.003
Aa23.wMel	0.006	<0.001
Aa23.wMelPop	0.2512	0.033

Table S1b: Optimal PLS-DA supervised model (DIMS), comprising of 70 forward selected *m/z* values only, built using 4 latent variables and tested using 1000 permutations. Forward selection maximises the statistical separation of groups by including only the highest ranked variables (*m/z* signals), and permutation testing indicates the validity of the model for this reduced dataset.

class	class error rate	p value
Aa23-T	0.0321	<0.001
Aa23.wMel	0.0009	<0.001
Aa23.wMelPop	0.0198	<0.001

Table S2a: PLS-DA supervised model of all three groups (LCMS), with all 4736 *m/z* values included, built using 5 latent variables and tested using 1000 permutations. These values indicate the validity of the model, highlighting the metabolic differences between the three classes.

class	class error rate	p value
Aa23-T	0.0152	<0.001
Aa23.wMel	0.0051	<0.001
Aa23.wMelPop	0.0360	<0.001

Table S2b: Optimal PLS-DA supervised model (LCMS), comprising of 500 forward selected *m/z* values only, built using 5 latent variables and tested using 1000 permutations. Forward selection maximises the statistical separation of groups by including only the highest ranked variables (*m/z* signals), and permutation testing indicates the validity of the model for this reduced dataset.

class	class error rate	p value
Aa23-T	0.0101	<0.001
Aa23.wMel	0.0063	<0.001

Aa23.wMelPop	0.0491	<0.001

Table S3a: PLS-DA supervised model of Aa23-T (control) versus infected (reclassified: Aa23.wMel + Aa23.wMel Pop) (DIMS), with all 2044 *m/z* values included, built using 5 latent variables and tested using 1000 permutations. These values indicate the validity of the model, highlighting the metabolic differences between the two classes.

**class**	**class error rate**	**p value**
Aa23-T (control)	0.1283	0.003
Infected	0.1305	0.003

Table S3b: Optimal PLS-DA supervised model (DIMS), comprising of 77 forward selected *m/z* values only, built using 5 latent variables and tested using 1000 permutations. Forward selection maximises the statistical separation of groups by including only the highest ranked variables (*m/z* signals), and permutation testing indicates the validity of the model for this reduced dataset.

**class**	**class error rate**	**p value**
Aa23-T	0.0183	0.001
Infected	0.0190	0.001

Table S4a: PLS-DA supervised model of Aa23-T (control) versus Aa23.wMel (DIMS), with all 2044 *m/z* values included, built using 2 latent variables and tested using 1000 permutations. These values indicate the validity of the model, highlighting the metabolic differences between the two classes.

**class**	**class error rate**	**p value**
Aa23-T	0.0165	0.001
Aa23.wMel	0.0165	0.001

Table S4b: Optimal PLS-DA supervised model (DIMS), comprising of 23 forward selected *m/z* values only, built using 2 latent variables and tested using 1000 permutations. Forward selection maximises the statistical separation of groups by including only the highest ranked variables (*m/z* signals), and permutation testing indicates the validity of the model for this reduced dataset.

**class**	**class error rate**	**p value**
Aa23-T	0.0001	<0.001
Aa23.wMel	0.0002	<0.001

Table S5a: PLS-DA supervised model of Aa23-T versus Aa23.wMelPop (DIMS), with all 2044 *m/z* values included, built using 3 latent variables and tested using 1000 permutations. These values indicate the validity of the model, highlighting the metabolic differences between the two classes.

**class**	**class error rate**	**p value**
Aa23-T	0.1971	0.020
Aa23.wMelPop	0.1979	0.020

Table S5b: Optimal PLS-DA supervised model (DIMS), comprising of 69 forward selected *m/z* values only, built using 3 latent variables and tested using 1000 permutations. Forward selection maximises the statistical separation of groups by including only the highest ranked variables (*m/z* signals), and permutation testing indicates the validity of the model for this reduced dataset.

**class**	**class error rate**	**p value**
Aa23-T	0.0456	0.002
Aa23.wMelPop	0.0445	0.002

Table S6a: PLS-DA supervised model of Aa23.wMel versus Aa23.wMelPop (DIMS), with all 2044 *m/z* values included, built using 2 latent variables and tested using 1000 permutations. These values indicate the validity of the model, highlighting the metabolic differences between the two classes.

**class**	**class error rate**	**p value**
Aa23.wMel	0.01375	0.005
Aa23.wMelPop	0.01025	0.005

Table S6b: Optimal PLS-DA supervised model (DIMS), comprising of 73 forward selected *m/z* values only, built using 2 latent variables and tested using 1000 permutations. Forward selection maximises the statistical separation of groups by including only the highest ranked variables (*m/z* signals), and permutation testing indicates the validity of the model for this reduced dataset.

**class**	**class error rate**	**p value**
Aa23.wMel	0.0030	0.002
Aa23.wMelPop	0.0009	0.002

Table S7a: PLS-DA supervised model of Aa23-T (control) versus infected (reclassified: Aa23.wMel + Aa23.wMel Pop) (LCMS), with all 4736 *m/z* values included, built using 2 latent variables and tested using 1000 permutations. These values indicate the validity of the model, highlighting the metabolic differences between the two classes.

class	class error rate	p value
Aa23-T	0.0160	<0.001
Infected	0.0160	<0.001

Table S7b: Optimal PLS-DA supervised model (LCMS), comprising of 463 forward selected *m/z* values only, built using 2 latent variables and tested using 1000 permutations. Forward selection maximises the statistical separation of groups by including only the highest ranked variables (*m/z* signals), and permutation testing indicates the validity of the model for this reduced dataset.

class	class error rate	p value
Aa23-T	0.0100	<0.001
Infected	0.0100	<0.001

Table S8a: PLS-DA supervised model of Aa23-T and Aa23.wMel (LCMS), with all 4736 *m/z* values included, built using 1 latent variable and tested using 1000 permutations. These values indicate the validity of the model, highlighting the metabolic differences between the two classes.

class	class error rate	p value
Aa23-T	0.0070	<0.001
Aa23.wMel	0.0068	<0.001

Table S8b: Optimal PLS-DA supervised model (LCMS), comprising of 59 forward selected *m/z* values only, built using 1 latent variable and tested using 1000 permutations. Forward selection maximises the statistical separation of groups by including only the highest ranked variables (*m/z* signals), and permutation testing indicates the validity of the model for this reduced dataset.

class	class error rate	p value
Aa23-T	0.0001	<0.001
Aa23.wMel	0.0004	<0.001

Table S9a: PLS-DA supervised model of Aa23-T versus Aa23.wMelPop (LCMS), with all 4736 *m/z* values included, built using 3 latent variables and tested using 1000 permutations. These values indicate the validity of the model, highlighting the metabolic differences between the two classes.

class	class error rate	p value
Aa23-T	0.0591	0.001
Aa23.wMelPop	0.0569	0.001

Table S9b: Optimal PLS-DA supervised model (LCMS), comprising of 235 forward selected *m/z* values only, built using 3 latent variables and tested using 1000 permutations. Forward selection maximises the statistical separation of groups by including only the highest ranked variables (*m/z* signals), and permutation testing indicates the validity of the model for this reduced dataset.

class	class error rate	p value
Aa23-T	0.0379	0.001
Aa23.wMelPop	0.0318	0.001

Table S10a: PLS-DA supervised model of Aa23.wMel versus Aa23.wMelPop (LCMS), with all 4736 *m/z* values included, built using 1 latent variable and tested using 1000 permutations. These values indicate the validity of the model, highlighting the metabolic differences between the two classes.

class	class error rate	p value
Aa23.wMel	0.0241	0.001
Aa23.wMelPop	0.0241	0.001

Table S10b: Optimal PLS-DA supervised model (LCMS), comprising of 57 forward selected *m/z* values only, built using 1 latent variable and tested using 1000 permutations. Forward selection maximises the statistical separation of groups by including only the highest ranked variables (*m/z* signals), and permutation testing indicates the validity of the model for this reduced dataset.

class	class error rate	p value
Aa23.wMel	0.0241	0.005
Aa23.wMelPop	0.0241	0.005

Table S11: LC-MS gradient. LC-MS separations were performed on a Dionex Acclaim C30 column (Thermo Scientific, 2.1 x 150 mm, 3 µm particles) and separated at 40°C with a flow rate of 200 µL/min and a gradient from solvent A (5mM ammonium acetate, 5 % isopropanol in water) over solvent B (5 mM ammonium acetate, 5 % isopropanol, 5 % water in acetonitrile) to solvent C (5 mM ammonium acetate, 5 % water in isopropanol).

Time [min]	A [%]	B [%]	C [%]	Flow [µl/min]
0.00	100	0	0	200
1.00	100	0	0	200
11.00	0	100	0	200
12.00	0	100	0	200
20.00	0	0	100	200
24.00	0	0	100	150
24.01	100	0	0	150
30.00	100	0	0	200

Table S12: XCMS / CAMERA script (in R) for processing cdf files

```
library(Hmisc)
library(xcms)
library(CAMERA)

xset <- xcmsSet(step=0.02,snthresh=5,mzdiff = 0.01)
grp <- group(xset,bw=10,mzwid = 0.05)
xsa <- annotate(grp, cor_eic_th=0)
peaklist<-getPeaklist(xsa)
write.csv(peaklist,'Sinkins_LCMSneg_a.csv')
```

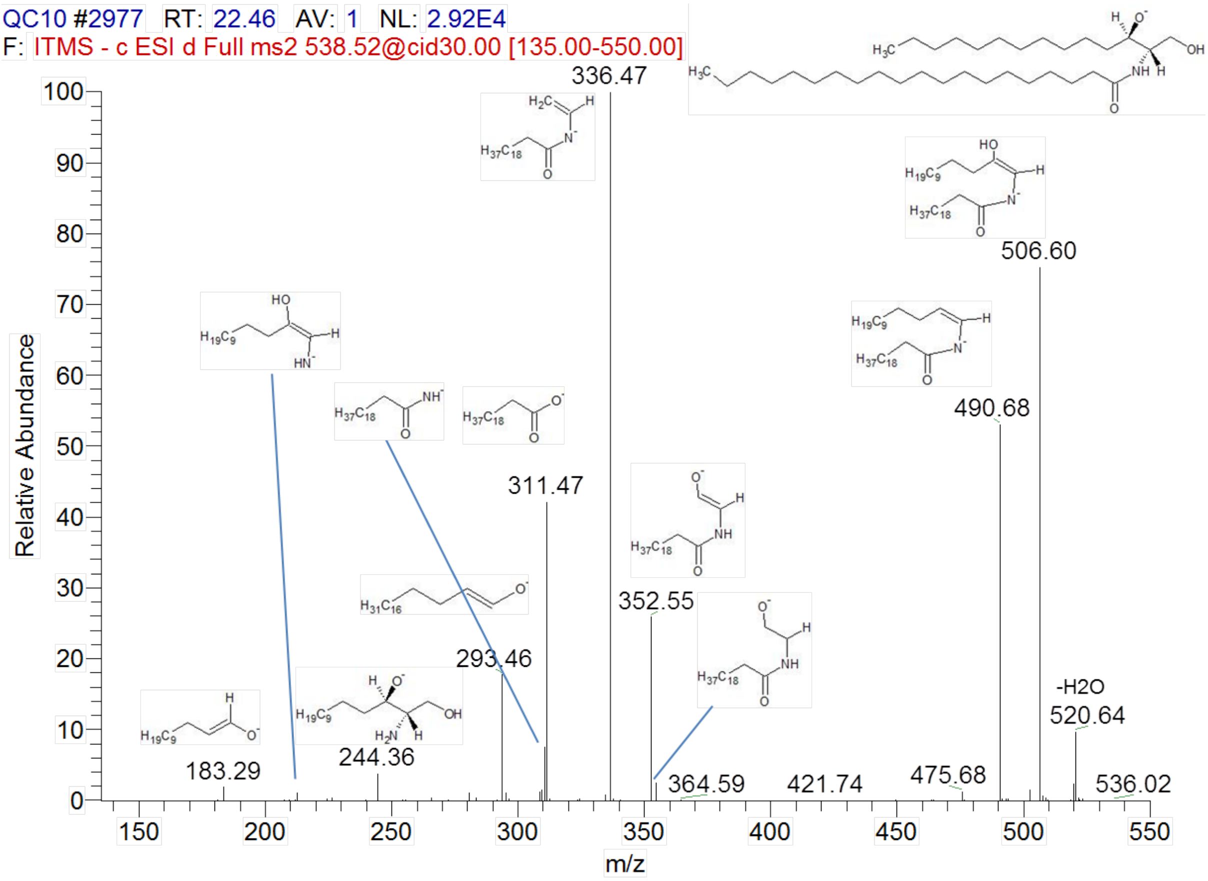

Fig S13: MS/MS spectrum of the ceramide signal at m/z 538 (Cer d14:0/20:0). The annotation follows the mechanism proposed by Hsu & Turk (1).

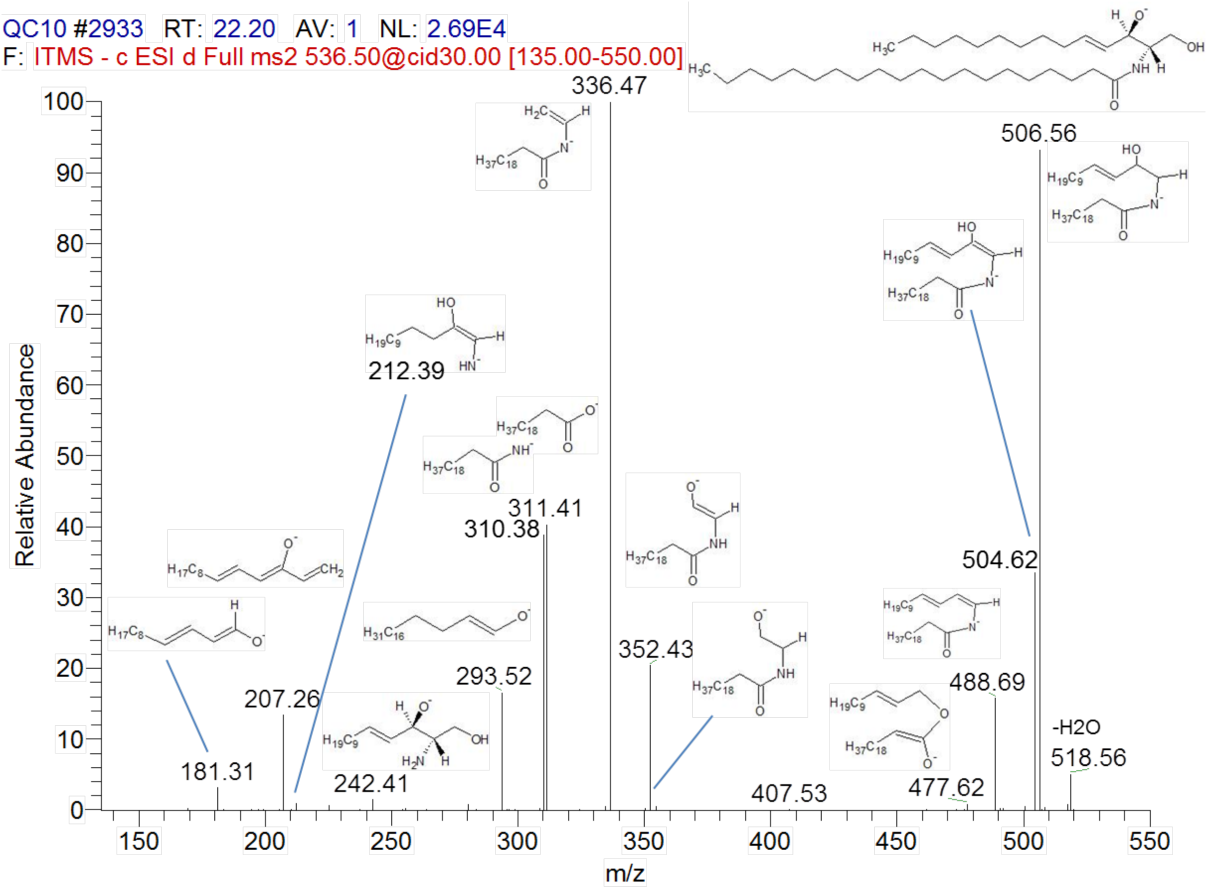

Fig S14: MS/MS spectrum of the ceramide signal at m/z 536 (Cer d14:1/20:0). The annotation follows the mechanism proposed by Hsu & Turk (2002).

S15 Table: Statistics and MS signal annotations for the DIMS experiments.

S16 Table: Statistics and MS signal annotations for the LC-MS experiments.

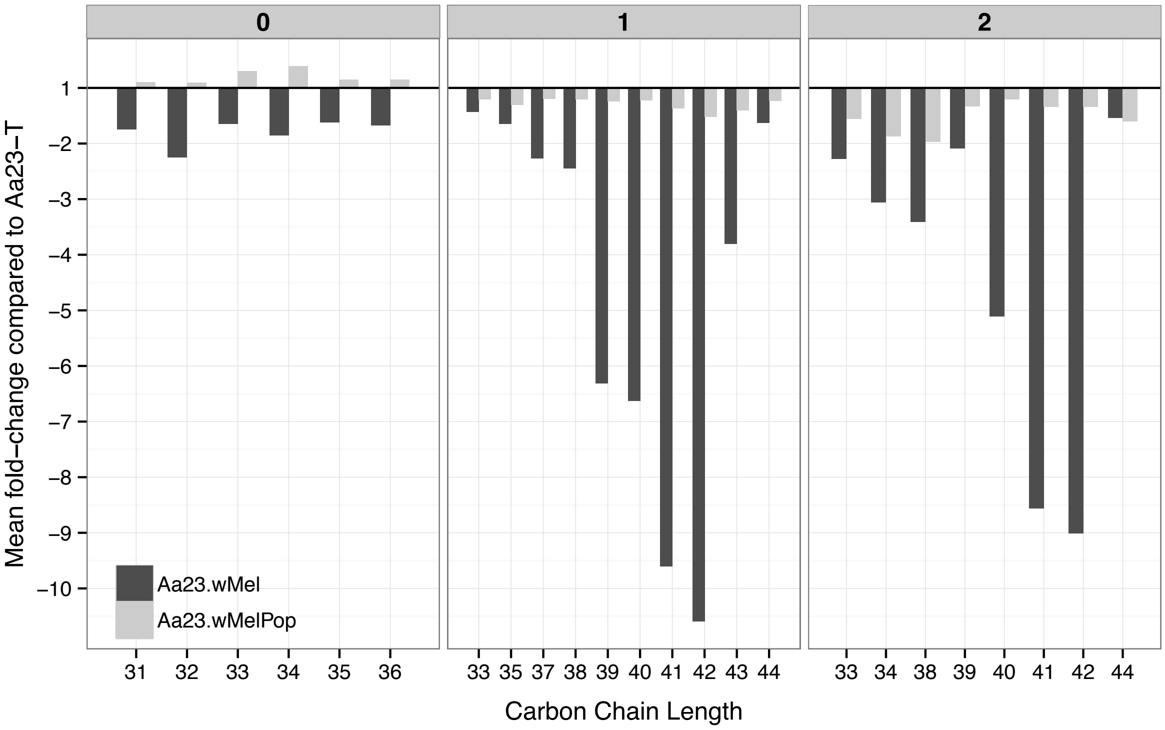

S17 Fig: Multiple ceramide species show strong decreases in *Wolbachia*-infected *Ae. albopictus* Aa23 cell lines.

Bar graphs showing mean fold-change in individual $[M+OAc]^-$ ion series ceramide species per level of saturation, ordered by carbon chain length. All species shown were significantly changed (ANOVA q < 0.05). **Aa23.*w*Mel:** relatively long chain species (Cer 42:2, 42:1, 40:1, 40:2, 41:1) displayed the largest changes (> 85 % decrease, *i.e.* around 10-fold) and somewhat shorter, sometimes saturated species (Cer 34:0, 35:1, 38:0, 38:1) displayed lesser changes (< 50%). **Aa23.*w*MelPop:** changes in abundance compared to Aa23-T differ in direction. The ten most decreased signals in the LC-MS dataset contained two or three double bonds, and in the DIMS dataset some unsaturated species (Cer 36:3, 38:3, 36:2, 33:1, and 35:1) were > 40% decreased.. However, > 30% increases were seen for 33:0, 34:0, and 38:0 saturated species in the DIMS data and for Cer 31:0 to 36:0 via LC-MS.

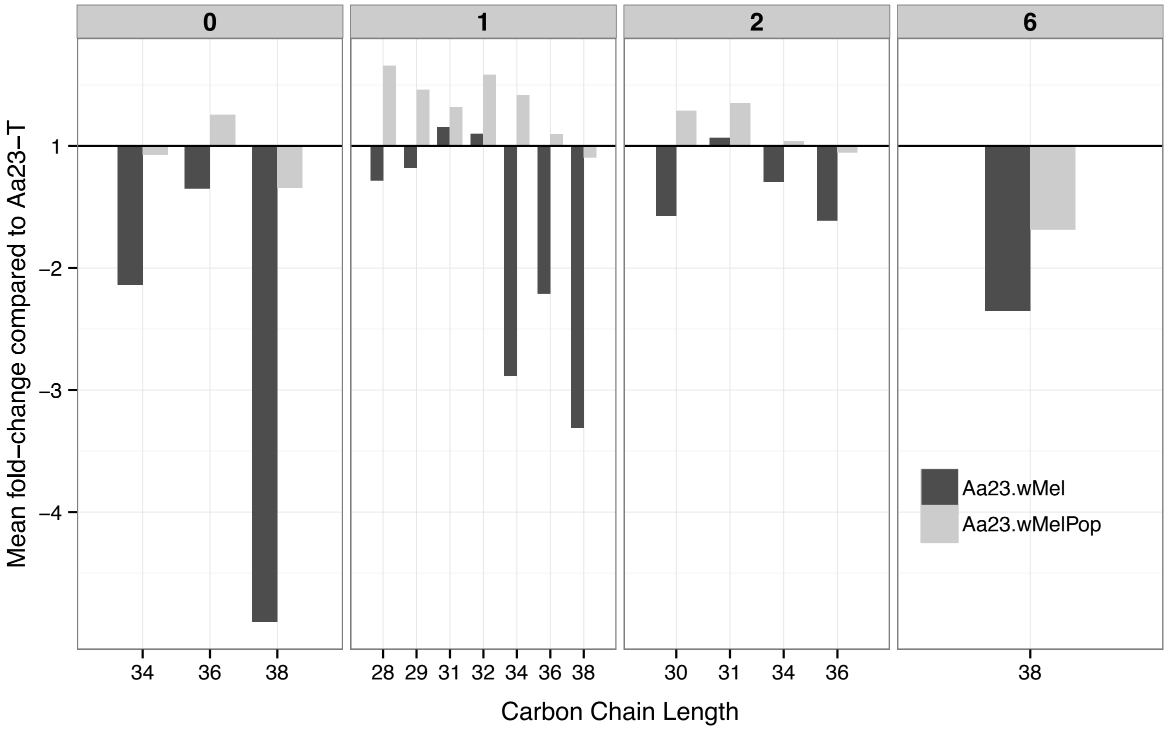

S18 Fig: Diacyglycerols (DGs) species in *Wolbachia*-infected *Ae. albopictus* Aa23 cell lines show both intensity decreases and increases.

Bar graphs showing mean % change (see description in Fig 3) in individual DG species per level of saturation, ordered by carbon chain length ($[M+OAc]^-$ ion series only). 0 = no double bonds (contains only saturated fatty acid species), 1 = one double bond (contains a monounsaturated fatty acid (MUFA), 2 = two double bonds. The strongest decreases in both cell lines were found for longer species (C34 to 38 total chain length). The largest increases were seen in DG 32:1, 31:1, and 31:2 for Aa23.*w*Mel and DG 28:1, 32:1, and 29:1 for Aa23.*w*MelPop.

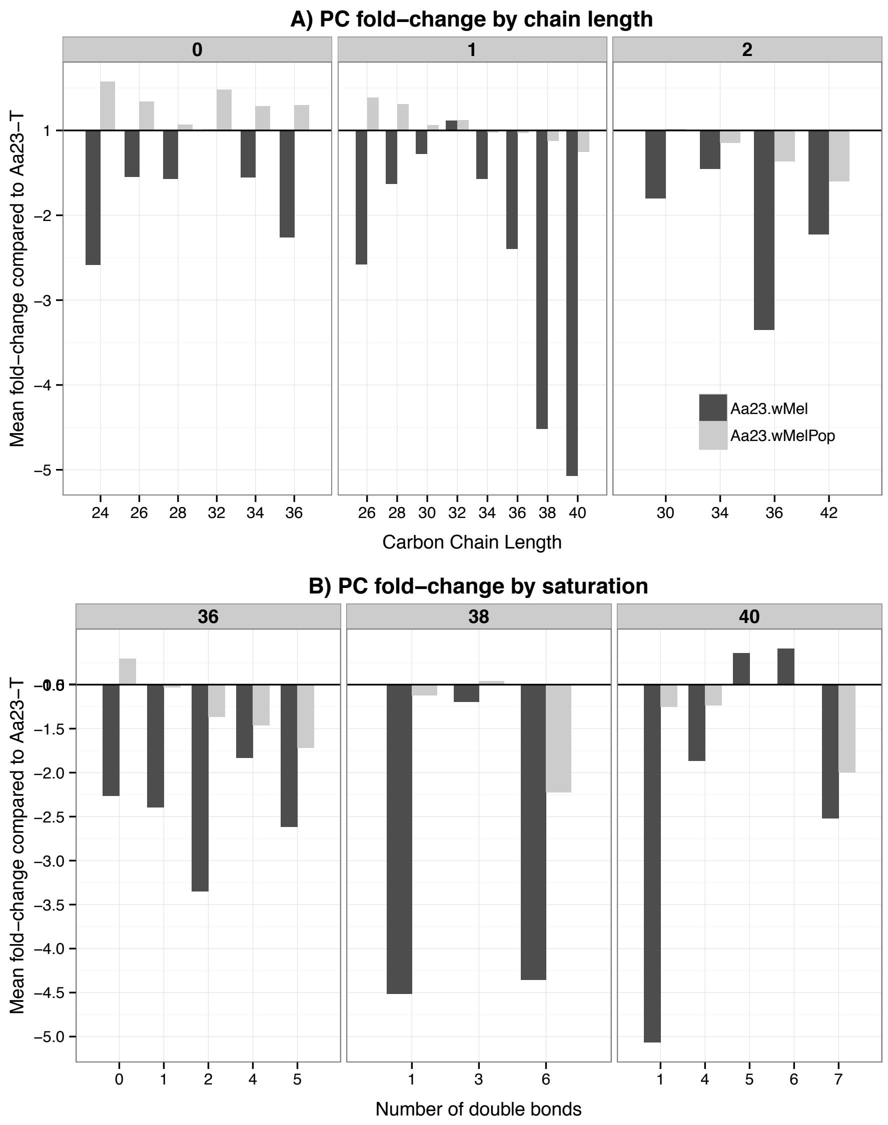

S19 Fig: Phosphatidylcholine (PC) species in *Wolbachia*-infected *Ae. albopictus* Aa23 cell lines show both intensity decreases and increases.

Bar graphs showing mean % change (see description in Fig 3) in individual PC species per level of saturation, ordered by carbon chain length ($[M+OAc]^-$ ion series only). 0 = no double bonds (contains only saturated fatty acid species), 1 = one double bond (contains a monounsaturated fatty acid (MUFA), 2 = two double bonds. **Aa23.*w*Mel:** Most species showed up to 80% decrease but five signals (PC 40:6, 40:5, 31:1, 32:1, 32:0) showed an increased abundance. **Aa23.*w*MelPop:** Longer-chain PCs (38:6, 40:7, 36:5, 35:2, 42:2) showed a > 50 % decrease while shorter-chain PCs (24:0, 32:0, 26:1, 26:0, and 28:1) showed a >30% increase.

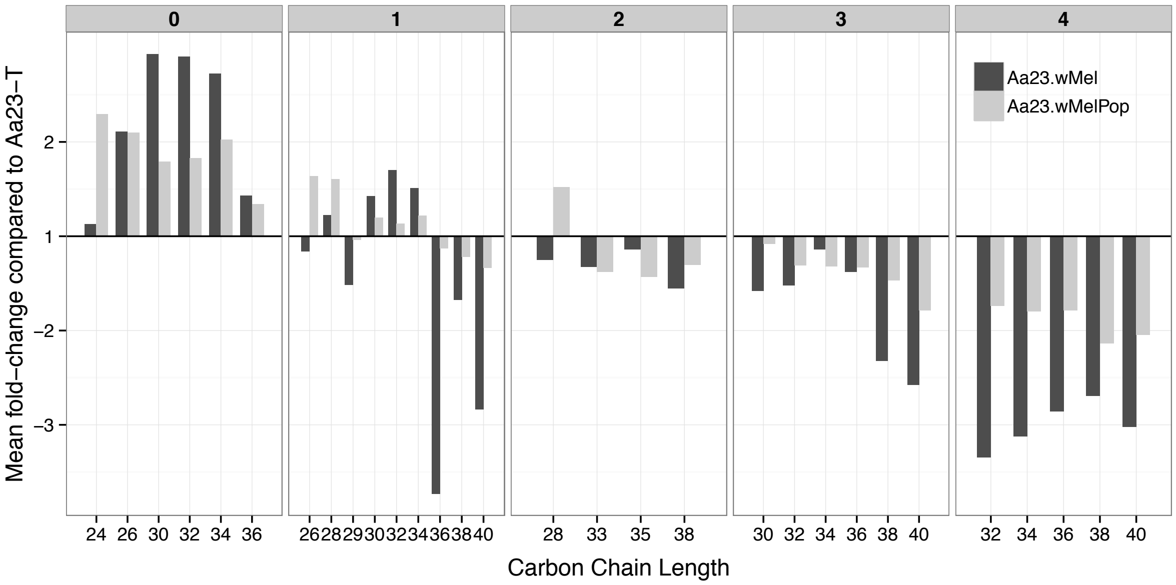

S20 Fig: Saturated phosphatidylethanolamine (PE) species increase and less saturated species decrease in *Wolbachia*-infected *Ae. albopictus* Aa23 cell lines.

Bar graphs showing mean % change (see description in Fig 3) in individual PE species per level of saturation, ordered by carbon chain length ([M-H]- ion series only). 0 = no double bonds (contains only saturated fatty acid species), 1 = one double bond (contains a monounsaturated fatty acid (MUFA), 2 = two double bonds. **Aa23.*w*Mel:** Unsaturated species with at least four double bonds were most reduced (10 species with > 65% reduction) and those with at least three double bonds constituted 14 out of 16 signals with a > 50% decrease while all 19 signals with a > 50% increase had less than or equal to one double bond, with the six largest increases representing saturated species. **Aa23.*w*MelPop:** Changes were very similar to Aa23.*w*Mel, with the 12 most decreased signals (> 40%) having at least three double bonds, and the nine most increased signals being saturated.

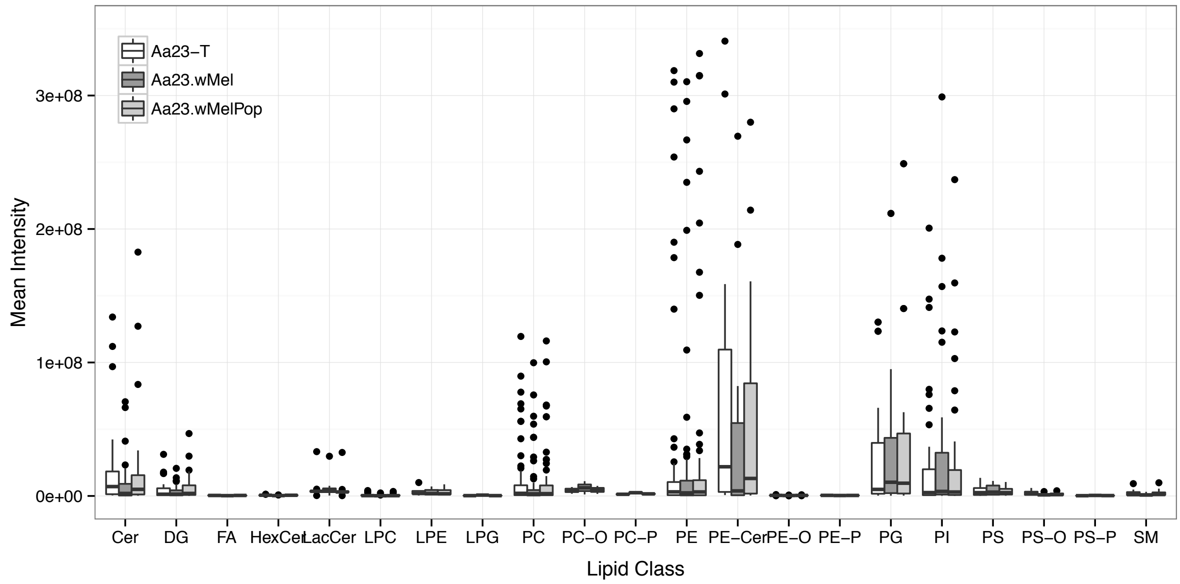

S21 Fig: Phosphatidylethanolamine (PE) and phosphatidylcholine (PC) lipid signals show highest intensity in LCMS analysis of *Ae. albopictus* Aa23-T cell lines

Box plot showing distribution of mean normalised signal intensity for each annotated lipid species, calculated across six biological replicates of each cell line and organised by lipid class. For clarity, six outliers have been excluded across the dominant PC and PE-Cer classes, where signal intensities ranged up to 7.5e+08. No absolute quantitative analysis was undertaken in this analysis so data is provided to illustrate broad relative patterns only.

References

1. **Hsu F-F, Turk J**. 2002. Characterization of Ceramides by Low Energy Collisional-Activated Dissociation Tandem Mass Spectrometry with Negative-ion Electrospray Ionization. J Am Soc Mass Spectrom **13**:558-570.
